# Synthesis of Fe-Doped Peroxidase Mimetic Nanozymes from Natural Hemoglobin for Colorimetric Biosensing and In Vitro Anticancer Effects

**DOI:** 10.3390/bios13060583

**Published:** 2023-05-27

**Authors:** Zahra Mohammadpour, Esfandyar Askari, Farhad Shokati, Hosna Sadat Hoseini, Mojtaba Kamankesh, Yasser Zare, Kyong Yop Rhee

**Affiliations:** 1 Biomaterials and Tissue Engineering Department, Breast Cancer Research Center, Motamed Cancer Institute, ACECR, Tehran 1517964311, Iran; esfandyaraskari@uvic.ca (E.A.); shokati@acecr.ac.ir (F.S.); hoseini.h.s@ut.ac.ir (H.S.H.); m.kamankesh@ut.ac.ir (M.K.); y.zare@aut.ac.ir (Y.Z.); 2 Department of Mechanical Engineering (BK21 Four), College of Engineering, Kyung Hee University, Yongin 17104, Republic of Korea

**Keywords:** reactive oxygen species, nanozyme, carbon dots, glucose, colorimetry

## Abstract

Despite their efficiency and specificity, the instability of natural enzymes in harsh conditions has inspired researchers to replace them with nanomaterials. In the present study, extracted hemoglobin from blood biowastes was hydrothermally converted to catalytically active carbon nanoparticles (BDNPs). Their application as nanozymes for the colorimetric biosensing of H_2_O_2_ and glucose and selective cancer cell-killing ability was demonstrated. Particles that were prepared at 100 °C (BDNP-100) showed the highest peroxidase mimetic activity, with Michaelis–Menten constants (K_m_) of 11.8 mM and 0.121 mM and maximum reaction rates (V_max_) of 8.56 × 10^−8^ mol L^−1^ s^−1^ and 0.538 × 10^−8^ mol L^−1^ s^−1^, for H_2_O_2_ and TMB, respectively. The cascade catalytic reactions, catalyzed by glucose oxidase and BDNP-100, served as the basis for the sensitive and selective colorimetric glucose determination. A linear range of 50–700 µM, a response time of 4 min, a limit of detection (3σ/N) of 40 µM, and a limit of quantification (10σ/N) of 134 µM was achieved. In addition, the reactive oxygen species (ROS)-generating ability of BDNP-100 was employed for evaluating its potential in cancer therapy. Human breast cancer cells (MCF-7), in the forms of monolayer cell cultures and 3D spheroids, were studied by MTT, apoptosis, and ROS assays. The in vitro cellular experiments showed dose-dependent cytotoxicity of BDNP-100 toward MCF-7 cells in the presence of 50 µM of exogenous H_2_O_2_. However, no obvious damage was induced to normal cells in the same experimental conditions, verifying the selective cancer cell-killing ability of BDNP-100.

## 1. Introduction

Natural enzymes are essential assets in the catalysis of vital biological reactions. Despite their efficiency and specificity, they are unstable under harsh physicochemical conditions and are also expensive. With the advent of nanotechnology, several research fields benefited from the extraordinary properties of nanosized objects [1,2,3,4,5,6]. In 2007, it was accidentally found that magnetic nanoparticles mimic the function of horseradish peroxidase (HRP), albeit showing higher robustness [7]. Further studies confirmed that, unlike natural enzymes, nanomaterials with enzyme-like activities (nanozymes) are prepared at low costs and large scales, their handling is more feasible, and their activity preserves under harsh conditions [8,9,10]. Nanozymes have been used extensively in environmental monitoring, sensing, in vivo imaging, and cancer therapy [2,11,12,13].

There is a wide collection of nanomaterials that reproduce the functions of natural enzymes [14,15,16,17,18,19]. The most studied aspect is the peroxidase-like activity of nanozymes, which is the catalytic conversion of hydrogen peroxide to water [20,21,22,23,24]. The reactive oxygen species (ROS) produced during such a reaction can attack redox substrates in the colorimetric assays of important (bio)analytes [25,26] or cause the degradation of organic pollutants [27]. In addition, peroxidase-mimicking nanozymes can kill cancer cells through oxidative stress induction [28,29]. In this respect, iron-containing nanozymes that function based on Fenton chemistry are one of the best peroxidase-mimicking nanozymes [30,31,32,33]. A widely used Fe-based nanozyme is the Fe_3_O_4_ nanoparticle, which suffers from low activity compared to natural enzymes due to an inherent tendency to aggregate [34]. To solve this problem, they are commonly decorated on 3D porous supports [35,36]. Fe-doped molecular organic frameworks (MOFs) possess a larger surface area than metal oxide nanozymes. However, they feature a low density of active sites due to the existence of high molecular weight organic ligands. Dong et al. conquered the problem by growing MIL-101(Fe) crystals on MoS_2_ nanosheets [37]. Alternatively, Mu et al. prepared bimetal-organic frameworks (Fe_x_Ni_y_-MOF) with enhanced peroxidase-like activity by introducing two metal ions into the synthetic precursors [38]. There are other breakthroughs made toward the development of Fe-based nanozymes, including the preparation of single iron site nanozymes [31,39]. The abovementioned catalysts are prepared using multiple precursors and laborious synthesis procedures, while some require organic solvents, all of which hinder the continuous development of Fe-based nanozymes.

The use of bioresources for the synthesis of Fe-doped nanozymes is rare in the literature. In the present study, we employed natural hemoglobin (Hb) as a single precursor to synthesize Fe-doped nanozymes. To this end, we extracted Hb from blood biowastes on large scales and converted them to Fe-doped nanozymes, which we call blood-derived nanoparticles (BDNPs), using a one-step hydrothermal process. To compare the performance of the BDNPs to Fe-doped nanozymes that are prepared via multiple precursors, we synthesized the Fe-doped nanozymes using citric acid and FeCl_2_ as the precursors [40] and demonstrated the contribution of metal ion microenvironments on nanozyme catalytic activities. Hb-derived BDNPs were employed for the colorimetric quantification of hydrogen peroxide and glucose. Furthermore, the therapeutic efficacy of BDNPs in the selective killing of cancer cells was studied.

## 2. Materials and Methods

### 2.1. Synthesis of BDNPs

The synthesis of BDNPs was accomplished according to the following procedure. An amount of 23 mL of the blood-derived Hb was mixed with 12 mL of deionized water in a 50 mL Teflon-lined stainless steel autoclave. The mixture was subjected to thermal treatment at 100 °C (BDNP-100), 125 °C (BDNP-125), 150 °C (BDNP-150), and 180 °C (BDNP-180) for 3 h. The mixture was allowed to cool down to room temperature. The raw product was washed with deionized water to remove unreacted Hb. To the solid mass, 5 mL of concentrated NaOH (5M) was added, followed by probe sonication for 10 min (with an amplitude of 8, pulse-on time of 1 s, and a pulse-off time of 1 s). The mixture was centrifuged at 12,000 rpm for 10 min. The supernatant was carefully collected and dialyzed using deionized water (48 h), followed by lyophilization.

### 2.2. Peroxidase Mimetic Property of BDNPs

A colorimetric approach was adopted to investigate the catalytic property of BDNPs. A solution of tetramethyl benzidine (TMB, 200 µM) and H_2_O_2_ (2 mM) in acetate buffer solution (50 mM, pH 5.1) was freshly prepared as the working solution before use. A total of 100 µL of the working solution was added to each well of the ELISA plate. The reaction was started by adding BDNPs to the final concentration of 27 ppb. An ELISA plate reader monitored the absorbance change in the wells at 630 nm for 30 min.

*Glucose assay.* Glucose oxidase and various concentrations of glucose were diluted with an acetate buffer solution (pH 5.0, 50 mM). A total of 44 µL of the mixture was added to each well of the ELISA plate reader, and the plate was incubated at 37 °C. After 30 min of incubation, a fixed volume of the colorimetric reagent comprising TMB and BDNP-100, which were premixed with acetate buffer, was added to each well, and the absorbance signal at 630 nm was recorded for 30 min. The final volume of the reaction mixture at each well was 200 µL, and the final concentrations of TMB and BDNP-100 were 200 µM and 0.262 mg/L, respectively. The absorbance value at t = 30 min was used for the analysis of the calibration and selectivity data.

Human serum and plasma were used as the real samples for the analysis of the glucose concentration through the standard addition method. Intravenous human blood was freshly collected, and the plasma was isolated by gradient centrifugation using Ficoll-Hypaque-1077 (GE health care-Sweden). The plasma sample was diluted with acetate buffer solution (50 mM, pH 5) by a 1:7 volume ratio, followed by the addition of glucose oxidase. Various concentrations of glucose were then added to equal volumes (44 µL) of diluted plasma and glucose oxidase, and the mixtures were incubated at 37 °C for 30 min. The addition of the colorimetric reagent and absorbance reading was conducted in the same way as mentioned above.

## 3. Results and Discussions

A hydrothermal procedure was adapted for the synthesis of the BDNPs [41,42]. The thermal treatment was conducted at four temperatures: 100 °C, 125 °C, 150 °C, and 180 °C. The precursor was the Hb biomolecules extracted from the blood biowastes. Hb was selected as the iron- and carbon-containing precursor for the synthesis of carbon nanoparticles. Based on the treatment temperature, the synthesized BDNPs were called BDNP-100, BDNP-125, BDNP-150, and BDNP-180. Based on the previous reports, we propose that in the hydrothermal reaction condition, high pressure and temperature denaturized the protein, followed by dehydration, pyrolysis, double bond formation, the emergence of aromatic centers, and nucleation, which finally resulted in the formation of fluorescent particles [43]. The temperature range in the present study covers all these steps. We aimed to investigate the physicochemical properties of the particles that were formed in this pathway. The particles with the highest catalytic activity and zero fluorescence (BDNP-100) to particles with zero catalytic activity and reasonable fluorescence (BDNP-180) were synthesized in the temperature range of 100–180 °C.

In the following sections, the physicochemical, spectral, and catalytic properties of the BDNP samples were studied, and their applications in sensor design and ROS-mediated cytotoxicity induction were elaborated.

### 3.1. Characterization

**Figure 1:**
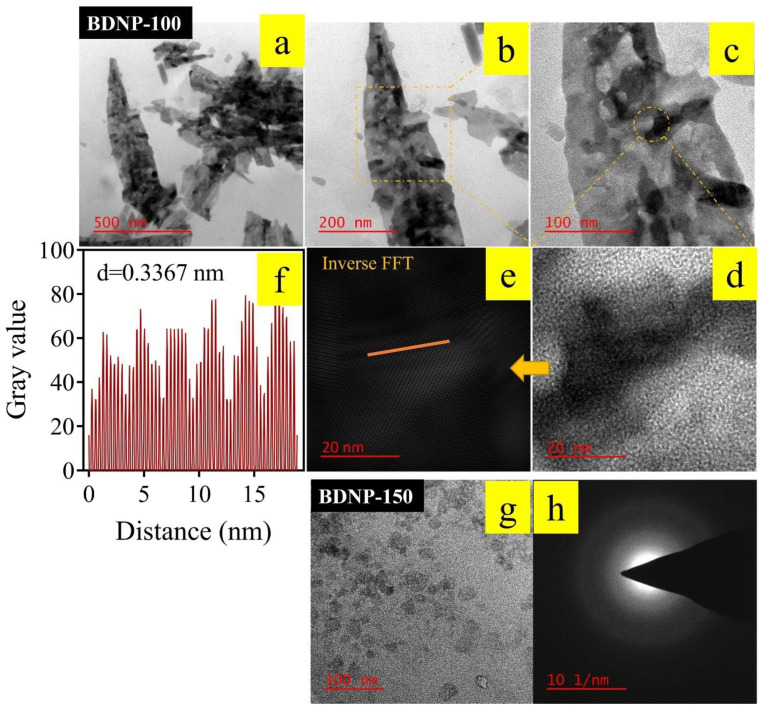
Morphological characterization of (**a**–**f**) BDNP-100 and (**g**,**h**) BDNP-150. Panels a–c show the sheet-like morphology of BDNP-100 with increasing magnification. Panels d and e are HRTEM images of the BDNP-100 particles before and after applying inverse FFT, respectively. (**f**) shows the height profile of the orange line shown in panel e. Panel g shows the semispherical morphology of BDNP-150 particles, and panel h is the SAED pattern of the BDNP-150 sample, signifying its lack of crystallinity.

TEM images of BDNP-100 and BDNP-150 are displayed in Figure 1. The samples’ size, morphology, and crystallinity are different. BDNP-100 comprises several small nanosheets overlaid on a few large carbon sheets. HRTEM data reveal the crystalline nature of BDNP-100. As the inverse FFT image shows, the interlayer spacing across the orange line was calculated as 0.3367 nm, close to the 002 planes of graphitic carbon. In addition, other interlayer distances of 0.21 nm and 0.26 nm were also found in the HRTEM images of BDNP-100, which is attributed to the 100 planes of graphite. In contrast, no crystallinity was observed for the BDNP-150 sample, possibly due to the destruction of the crystalline texture at higher hydrothermal temperatures [44]. The particles in BDNP-150 are semispherical, with an average diameter of 19.7 nm.

**Figure 2:**
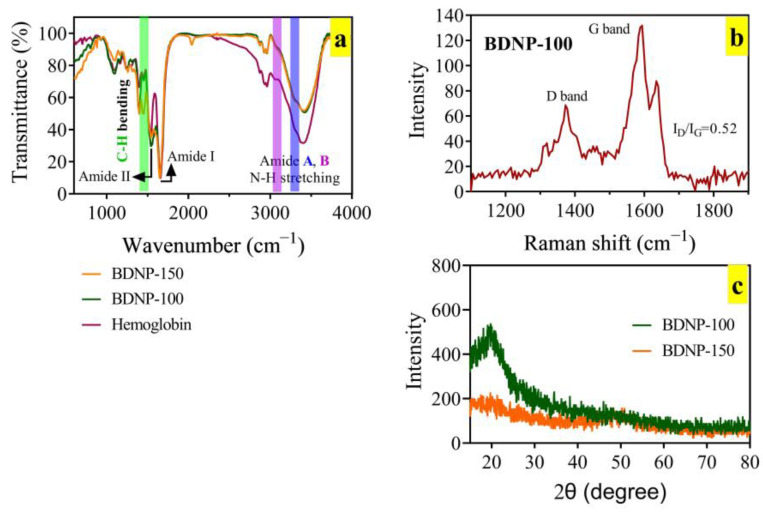
(**a**) FTIR, (**b**) Raman and (**c**) XRD patterns of BDNP samples. In panel a, the FTIR spectrum of hemoglobin is presented for comparison.

FTIR spectroscopy was employed for the evaluation of the structural change in Hb during the thermal treatment (Figure 2a). The peak position of the amide I band of Hb, originally located at 1659 cm^−1^, shifted to 1651.40 cm^−1^ and 1652.49 cm^−1^ for BDNP-100 and BDNP-150, respectively. The frequency shift implies that hydrothermal treatment induces protein conformational change [45,46]. Furthermore, the intensity of amide II was reduced in BDNP-150 compared to BDNP-100, which contributed to protein structural denaturation, as reported in previous studies [47]. BDNP-150 exhibited a substantial increase in the intensity of C-H bending (υ~1450 cm^−1^) compared to the Hb and BDNP-100. According to previous reports, the reason for such an intensity change is inconclusive. What is mutual, however, is that in different studies, the change in the intensity of C-H bending is assigned to the change in the interior hydrophobic interactions and the extent of the hydrophobic groups’ exposure to the polar environment [48]. The intensity of C-N (1543 cm^−1^) and C-O (1100 cm^−1^) decreased upon the temperature increase in the hydrothermal process, and double bonds started to form, as designated by the emergence of a sharp peak at 2035 cm^−1^. FTIR spectra reveal a more intense structural change in BDNP-150 than in BDNP-100.

XRD and Raman spectroscopy were employed for the crystallinity evaluation of the samples. The Raman spectrum of BDNP-100 in Figure 2b exhibits the characteristic D- and G-bands at 1372 and 1594 cm^−1^ and are associated with sp3 and sp2 carbons, respectively. Carbon materials, in which the intensity ratio of the D-to-G band is less than one, are identified to be less defective materials with a high degree of graphitization [49]. Our Raman results confirmed the presence of graphitized and defective sites in the sample. However, the I_D_/I_G_ ratio of less than unity confirms the high degree of graphitization of BDNP-100. The existence of ordered and disordered carbon in the Raman spectrum was reflected in the XRD pattern of the sample (Figure 2c). We presume that a broad peak, located at 2θ~20°, may have arisen from the fact that the XRD collected information on the graphitized and disordered particles at the same time. Compared to BDNP-100, BDNP-150 does not show any pattern in its XRD spectrum, demonstrating zero crystallinity, which is in accordance with the HRTEM data. It is noteworthy that HRTEM evaluates a local region of the sample, while XRD spectroscopy screens a larger portion of the sample.

The elemental composition of BDNP-100 and BDNP-150 was obtained from EDS, ICP-MS, and XPS analyses. According to the XPS results, there are plenty of hydrophilic functional groups in the samples, demonstrating a high solubility of nanoparticles in water. Figures S1 and S2 show that the chemical composition of both samples was almost similar, with no signal associated with the iron atoms observed, which was a result of iron scarcity in native and denatured hemoglobin biomolecules, compared with C, N, and O. Likewise, the amount of iron detected by EDS mapping was very low (Figure S3). Similar to our study, the XPS peak of Fe in hemoglobin was not detected in previous reports [50,51] because of the low concentration of Fe in hemoglobin. However, we detected iron atoms in the ICP-MS analysis of the samples (Table 1).

Deconvolution of the high-resolution XPS of C1s and O1s revealed a decrease in the amount of C-O bonds and an increase in the amount of C=O bonds in BDNP-150 compared to BDNP-100. The elemental analysis also revealed a higher O/C ratio in BDNP-150 (0.22) than in BDNP-100 (0.16), indicating higher oxygenated edge groups due to the scissoring and delamination of large graphitic sheets. The same trend was observed in the EDS results. The contribution of C=C in the C1s spectrum of BDNP-150 (29.77%) was higher than that of BDNP-100 (27.35%). Furthermore, a peak associated with graphitic nitrogen appears in the N1s spectrum of BDNP-150, unlike BDNP-100, signifying nitrogen doping in the former particles [52,53]. The sulfur signal in BDNP-100 was barely visible, and no detectable sulfur signal was observed in BDNP-150. The reason for such an observation is possibly due to a significant change in the chemical status of sulfur upon protein denaturation, as reported previously [54].

According to the characterization data of native hemoglobin and the hydrothermally treated samples, it is deduced that the molecular precursor carbonizes after hydrothermal treatment at 100 °C. The product (BDNP-100) comprises carbon sheets with a wide range of lateral sizes. The sheets are highly crystalline, similar to graphite. Further, an increase in the temperature results in the scissoring of larger sheets and thus transforms them into smaller semispherical particles. The size difference between BDNP-100 and BDNP-150 explains their distinct optical properties, which are elaborately presented in the supporting information (Figure S4).

### 3.2. Catalytic Properties of BDNP Samples

**Figure 3:**
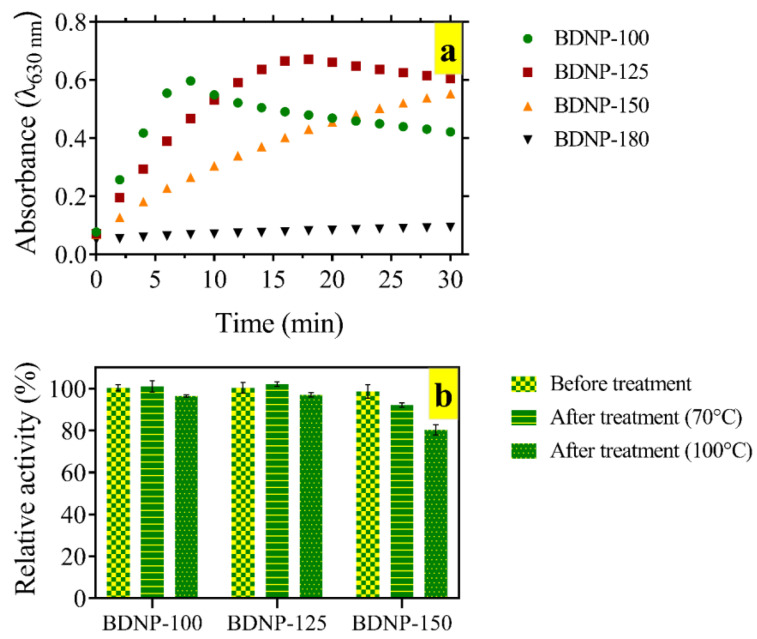
(**a**) The UV-Vis spectral change in oxidized TMB over time in the presence of BDNP samples. The reaction proceeded in 1 mL of acetate buffer (50 mM, pH 5) containing 200 µM TMB, 2 mM H_2_O_2_ and 27 ppb BDNP. (**b**) Stability of the catalytic activity after heating the catalyst suspension to 70 °C and 100 °C.

The catalytic activity of BDNP-100, BDNP-125, BDNP-150, and BDNP-180 toward the H_2_O_2_ reduction was evaluated by a TMB-based colorimetric assay. The catalyst concentration was adjusted in such a way that the amount of iron in all BDNP samples was identical. Figure 3a shows the absorbance change at 630 nm versus time. The colorimetric signal in the presence of BDNP-100 raised rapidly and reached a maximum value within 8 min. With the same amount of iron, the rate of signal change decreased for BDNP-125 and BDNP-150 and reached almost zero for BDNP-180.

As BDNP-180 did not show any colorimetric signal, they were excluded from the catalytic activity evaluation. The catalytic stability of the BDNP samples in harsh thermal conditions was studied. To this end, the samples were subjected to heating at 70 °C and 100 °C (Figure 3b). After cooling them down to room temperature, the catalytic activity was compared to the untreated samples (control). We observed that BDNP-100 and BDNP-125 retained their activity to almost 100% after heating to 70 °C and lost only 3–4% of their activity after heating to 100 °C. On the other hand, BDNP-150 lost its catalytic activity by 8% and 17% after treatment at 70 °C and 100 °C, respectively.

Therefore, among the synthesized samples, BDNP-100 was the best for catalyzing the H_2_O_2_ reduction and being used in sensing applications (H_2_O_2_ and H_2_O_2_-related targets). However, it had no fluorescence signal observed under the UV light. BDNP-150 exhibits lower catalytic activity and stability compared to BDNP-100; however, it was fluorescent. Hence, BDNP-150 can be used both as a peroxidase mimetic and a fluorescent probe. BDNP-180 was fluorescent and did not show any peroxidase-like properties; thus, it can be used only as a fluorescent probe.

**Figure 4:**
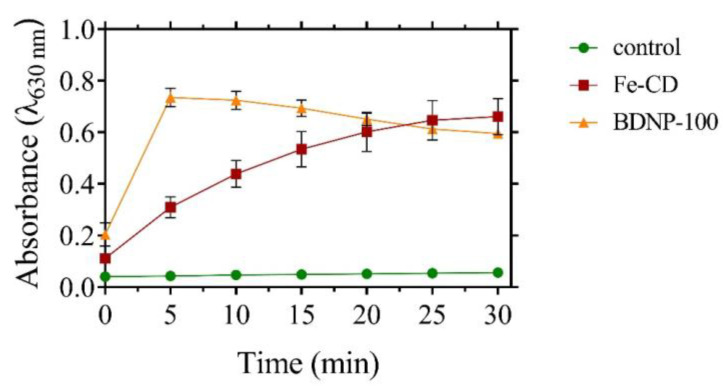
Comparison of the catalytic activity of BDNP-100 and Fe-CDs toward TMB oxidation in the presence of H_2_O_2_. The reaction buffer was acetate buffer (50 mM, pH 5) containing 200 µM TMB and 2 mM H_2_O_2_. The final concentration of catalysts was the same as 47 ppb.

We also compared the peroxidase mimetic activity of BDNP-100 to the Fe-doped nanozymes prepared by citric acid as the carbon source and FeCl_2_.4H_2_O as the dopant (Fe-CDs) [40]. Over the same concentration of catalysts, we found that BDNPs exhibit higher peroxidase mimetic activity, as shown in Figure 4. We measured the concentration of the iron atoms in the samples using ICP and achieved a Fe_(BDNPs)_/Fe_(Fe-CD)_ ratio of 0.148. Despite the low amount of iron in BDNP-100 compared to the Fe-CDs, the catalytic activity of the former was considerably higher. We propose that the chemical microenvironment around the metal ion in BDNP-100 is structurally closer to the microenvironment of Hb (as the precursor) than that of Fe-CD, which is synthesized using citric acid and FeCl_2_ as precursors. The inferior catalytic activity of Fe-CD than BDNP-100 is demonstrated by comparing their kinetic parameters, as will be discussed in Section 3.3. The V_max_ toward H_2_O_2_ reduction was lower in Fe-CD compared to the BDNP or Hb samples, which agrees well with the lower catalytic activity of Fe-CDs in Figure 4. The K_m_ value of Fe-CD for H_2_O_2_ was lower than BDNP-100 and BDNP-150, demonstrating a higher affinity of Fe-CD than BDNPs toward H_2_O_2_.

### 3.3. Kinetic Study

The effect of the synthesized temperature on the catalytic efficiency of the BDNP samples was evaluated using Michaelis–Menten kinetics. Nonlinear regression was employed for the calculation of K_m_ and V_max_, and was directly based on the Michaelis–Menten kinetic model. The respected values are presented in Table 2. The kinetic parameters of lyophilized commercial Hb (Sigma), dated Hb extracted from blood, and freshly extracted Hb from blood were also calculated as the reference, and the results were compared to those obtained for BDNP-100 and BDNP-150. Interestingly, regardless of the liquid or solid states of the initial Hb sample, the affinity toward a certain substrate is identical. For example, the K_m_ value of the Hb samples for H_2_O_2_ is over the range of 1.54–2 mM, while the K_m_ value for TMB is in the concentration range of 0.142–0.291 mM. The results reveal that the affinity of the Hb samples toward TMB is higher than that of H_2_O_2_. The difference between Hb samples is reflected in the V_max_ values. The maximum reaction rate between TMB and H_2_O_2_ is achieved in the presence of the Hb extracted from fresh blood. The Hb sample kept at 4 °C for a month displayed a lower reaction rate than the fresh Hb, possibly due to the partial deformation of the protein structure. The V_max_ value for the lyophilized Hb was considerably lower than the liquid states of Hb. Analyses of the UV-Vis spectra of the Hb samples showed that the iron oxidation state in the blood-extracted Hb in the liquid state is ferrous, while the iron oxidation state in the lyophilized Hb (Sigma) is ferric (Figure S5) [55]. It is well-accepted that ferrous iron is more active than ferric iron in catalytic H_2_O_2_ reductions [56].

The K_m_ value of the BDNPs toward TMB is very close to the values obtained for the Hb samples. In contrast, the affinity of the BDNPs toward H_2_O_2_ was less than the Hb samples. It is proposed that the active site of the Hb that is responsible for the attachment of H_2_O_2_ is altered considerably during the hydrothermal treatment, while the structural change in the TMB-attached site may not change significantly. In addition, the maximum reaction rate of the H_2_O_2_ reduction and TMB oxidation in the presence of BDNP-100 was 2.7 and 2.1 times higher than that of BDNP-150. Although the kinetic study reveals that the enzymatic activity of Hb is superior to the BDNP samples, thermal instability and the loss of catalytic activity at higher temperatures (Section 3.4) make Hb unfavored in practical applications.

### 3.4. Optimization and Robustness Evaluation

**Figure 5:**
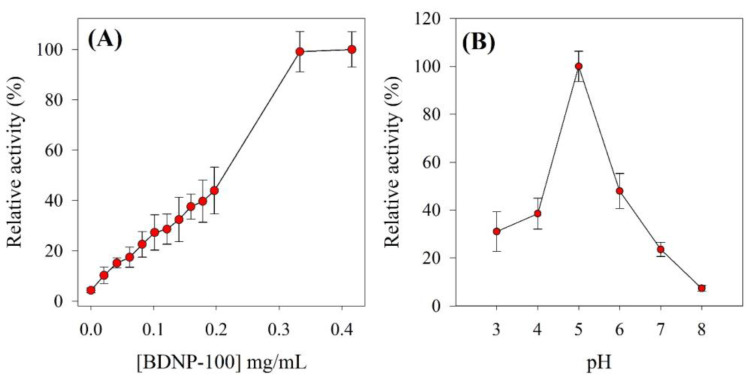
Colorimetric response of H_2_O_2_ in the presence of (**A**) variable concentrations of BDNP-100 and (**B**) pHs. In (**A**), TMB = 200 µM, H_2_O_2_ = 0.5 mM, pH 5.0; in (**B**), TMB = 200 µM, H_2_O_2_ = 5 mM, BDNP-100 = 0.031 mg/L.

In light of the higher efficiency of BDNP-100 than BDNP-150 toward H_2_O_2_ reduction, optimization experiments proceeded using the former catalyst. The effect of BDNP’s concentration and pH on the progression of the catalytic reduction of H_2_O_2_ was investigated. In each case, relative activity was measured, which is defined as the ratio of the oxidized TMB absorbance in the presence of H_2_O_2_+BDNP to the absorbance in the presence of H_2_O_2_ alone. Figure 5A shows that a higher amount of BDNP in the reaction mixture resulted in higher relative activity until a plateau was reached over the concentration range of 0.3–0.4 mg mL^−1^. Figure 5B illustrates that the highest relative activity of BDNP-100 was observed at pH 5. A decrease in the activity at alkaline pHs is possibly due to the instability of H_2_O_2_ at high pH values.

**Figure 6:**
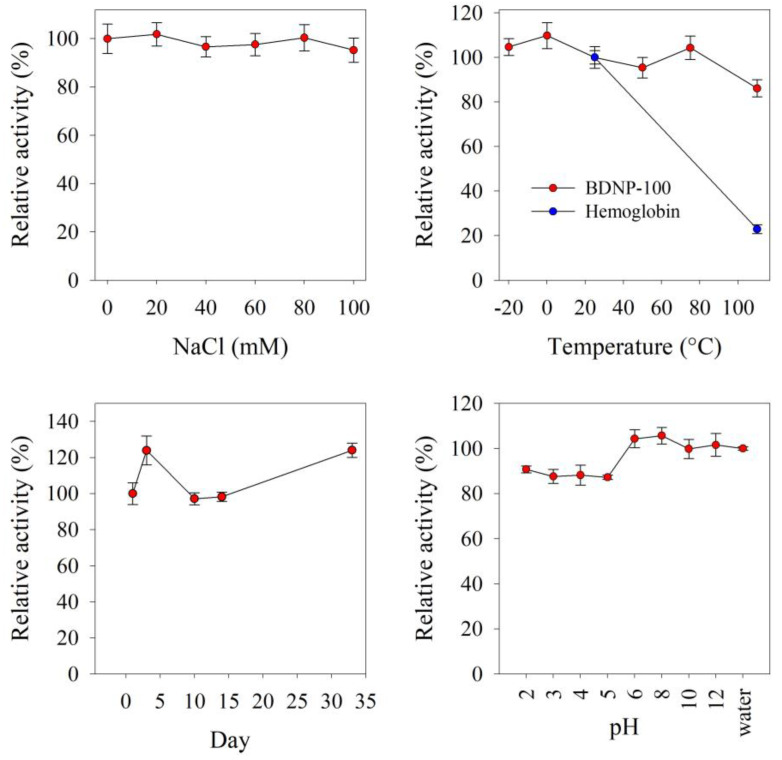
The effects of storage conditions, including NaCl concentration (20–100 mM), pH (2.0–12.0), temperature (−20–110 °C), and time (33 days) on the relative activity of BDNP-100.

One advantage of using nanozymes instead of enzymes in catalytic assays is the higher stability of the former against harsh conditions. The peroxidase-like activity of BDNP-100 was investigated at different storage conditions, including the pH, temperature, and ionic strength. As shown in Figure 6, ionic strength does not considerably affect the catalytic activity. The effect of a wide range of storage temperatures on the relative activity was explored. BDNPs’ activity was intact at low temperatures down to −20 °C and retained 86% of its initial activity at 110 °C. Contrarily, the Hb lost 77% of its activity at high temperatures, which is expected and thus considered a drawback of the natural proteins. The effect of the solution pH on the activity of BDNPs was also investigated. At low pH values, the activity was lower than that of higher pH values and water. We expect that the BDNPs become protonated at low pHs, making them more susceptible to aggregation. As a result, their activity decreases. Changes in the catalytic activity over time showed that, over 33 days, the catalytic activity fluctuated by 24%. Altogether, the results in Figure 6 demonstrate the robustness of BDNP-100 as a catalytic nanomaterial.

### 3.5. H_2_O_2_ Assay

**Figure 7:**
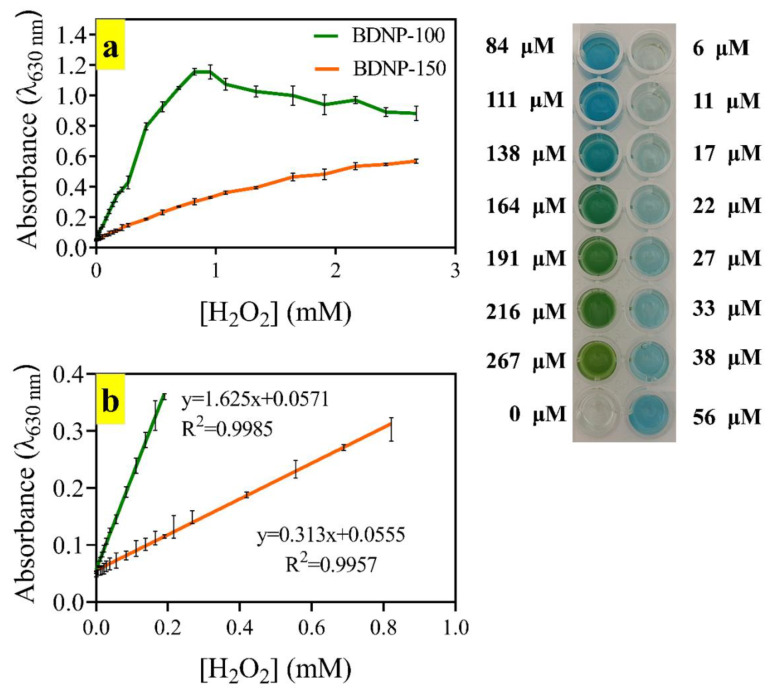
(**a**) Dynamic and (**b**) linear colorimetric response of BDNP-100 and BDNP-150 to H_2_O_2_. TMB = 200 µM, BDNPs = 0.262 mg/L, acetate buffer (pH 5, 50 mM). The digital picture of the ELISA plate was taken 30 min after the reaction started.

We further evaluated the sensitivity of BDNP-100 and BDNP-150 against different concentrations of H_2_O_2_ (Figure 7). As expected, the slope of the calibration plot when using BDNP-100 as the catalyst was higher than that of BDNP-150. All absorbance data were recorded at 630 nm using an ELISA plate reader 10 min after the reaction commenced. At H_2_O_2_ concentrations higher than 1 mM, the second step of catalytic TMB oxidation is triggered. Thus, the absorbance value at 630 nm decreased, and the solution’s blue color changed to green [26].

In contrast, the blue color slowly evolved during the TMB oxidation by BDNP-150, i.e., the first oxidation step prevails during the test period. The observation coincides with the kinetic studies, in which BDNP-100 was found to be more reactive toward both substrates than BDNP-150. The linear range for the H_2_O_2_ assay was 0–0.19 mM and 0–0.82 mM for BDNP-100 and BDNP-150, respectively. The limit of detection (LOD) for the colorimetric determination of H_2_O_2_ by BDNP-100 and BDNP-150 was calculated as 6 µM and 49 µM, respectively.

**Figure 8:**
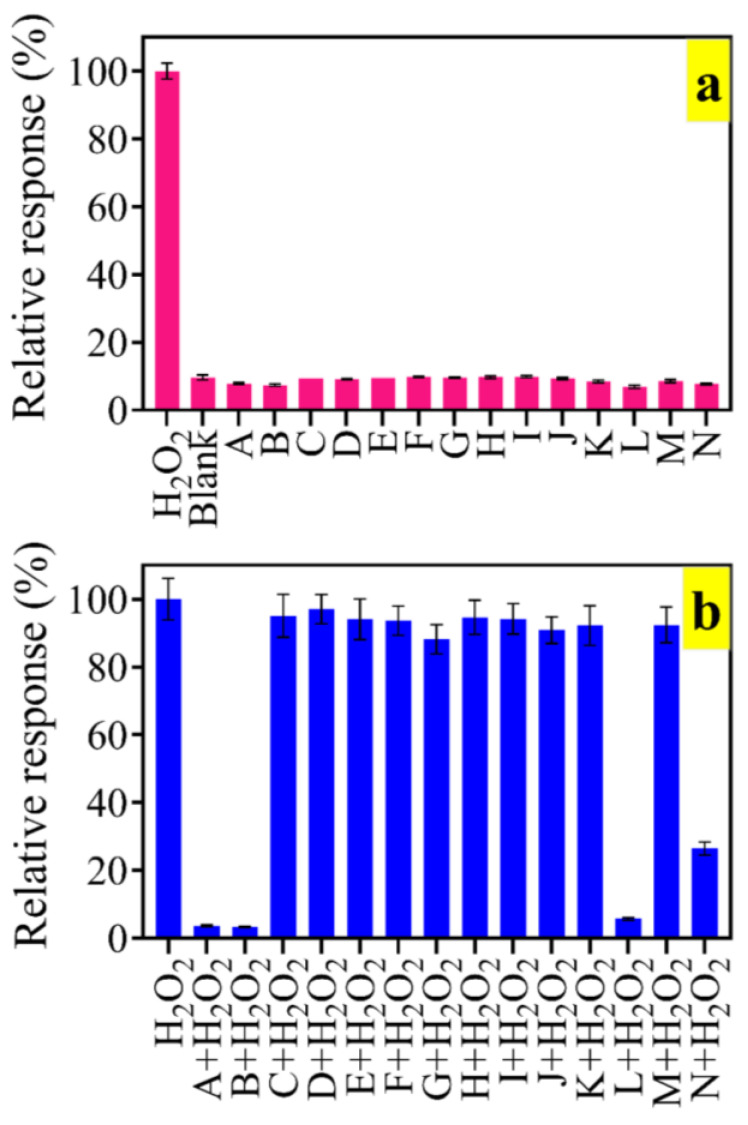
Selectivity of the colorimetric assay (**a**) in the absence and (**b**) in the presence of H_2_O_2_. A: cysteine, B: ascorbic acid, C: serine, D: asparagine, E: NaCl, F: MgCl_2_, G: tryptophan, H: KCl, I: arginine, J: methionine, K: leucine, L: glutathione, M: glucose, N: uric acid; TMB = 200 µM, H_2_O_2_ = 0.5 mM and other species were 1.17 mM, pH 5; BDNP-100 = 0.262 mg/L.

The selectivity of the present colorimetric assay for the quantification of H_2_O_2_ was studied. The colorimetric signal in the presence of the interference alone (Figure 8a) showed that none of the species increased the absorbance value except H_2_O_2_. A further evaluation of the TMB oxidation by H_2_O_2_ that was coexistent with interfering ions and molecules (Figure 8b) revealed that cysteine, ascorbic acid, glutathione, and partially, uric acid, prevented color generation. In our previous reports, we demonstrated that cysteine and glutathione are radical scavengers via their sulfhydryl groups [25,26]. Ascorbic acid, however, may either induce ROS generation or act as an antioxidant, depending on its concentration [57]. In the present study, the ascorbic acid concentration was 1.17 mM, which is high enough for the antioxidant role to prevail.

### 3.6. Glucose Assay

**Figure 9:**
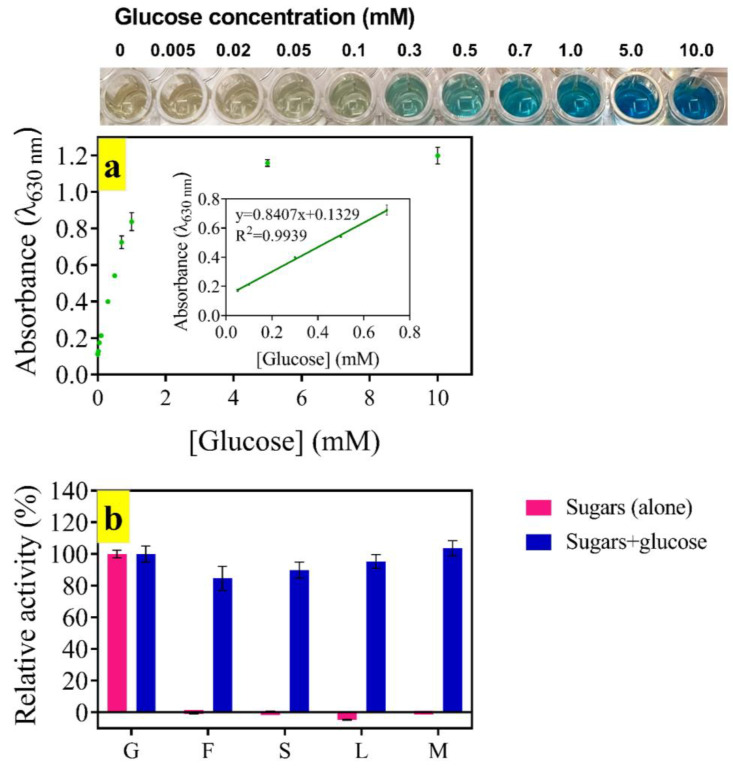
(**a**) Calibration plot of glucose; (**b**) selectivity of the colorimetric assay toward different sugars presented in the sample individually (red bars) and co-existed with glucose (gray bars). G: glucose, F: fructose, S: sucrose, L: lactose, M: maltose.

BDNP-100, as a peroxidase mimetic nanozyme, was combined with glucose oxidase and employed for glucose detection through a cascade catalytic reaction. In the presence of molecular oxygen, glucose oxidase converts glucose to gluconic acid and H_2_O_2_ at 37 °C. BDNP-100 catalyzes the TMB oxidation via H_2_O_2_ and produces a blue color. Higher levels of glucose generate more colored oxidation products and higher absorbance values (Figure 9a). The relation of the signal to the concentration was linear over the range of 0–0.7 mM. The LOD (3σ/N) and LOQ (10σ/N) were calculated as 40 µM and 134 µM, respectively (Figure 9a, inset). The response time of the proposed colorimetric assay was 4 min, and it was selective toward glucose. Other sugars, including fructose or lactose, did not react with glucose oxidase to generate H_2_O_2_ (Figure 9b). The co-existence of the possible interfering species with glucose was also studied. Statistical t-tests showed that the activity difference in the presence of glucose and glucose co-existed with fructose and was not significant at a 2% confidence level (t = 2.92, degrees of freedom = 4). The responses to glucose and glucose co-existed with sucrose, lactose, and maltose and were not significantly different at a 5% level (t = 2.48, degrees of freedom = 4). The linear range of our probe is wider than most of the reported assays, and the response time is shorter (Table S1). Although the LOD value is comparable to or higher than the reported assays, the sensitivity of our assay is good enough for the determination of fasting blood sugar in the serum samples of healthy (3.9–5.6 mM) and diabetic (7.0 mM or above) people. The obtained results show that a quantitative and selective detection of glucose with the present colorimetric assay is feasible.

### 3.7. Real Sample Analysis

The glucose levels of fresh human serum and plasma, used as the real samples, were measured by the proposed assay, and the results were compared to those measured by a clinical laboratory. The results presenting the accuracy and precision of the assay are shown in Table 3. Various glucose concentrations were spiked to the real sample, and the recovery values were calculated over the range of 95–110%. Additionally, the glucose level in the unspiked sample was close to that measured by the gold standard approach. The amount of relative error spanned over the range of 1.46–8.71%, showing the acceptable accuracy of the present system.

### 3.8. Biological Characterization of BDNP-100

#### 3.8.1. MTT

**Figure 10:**
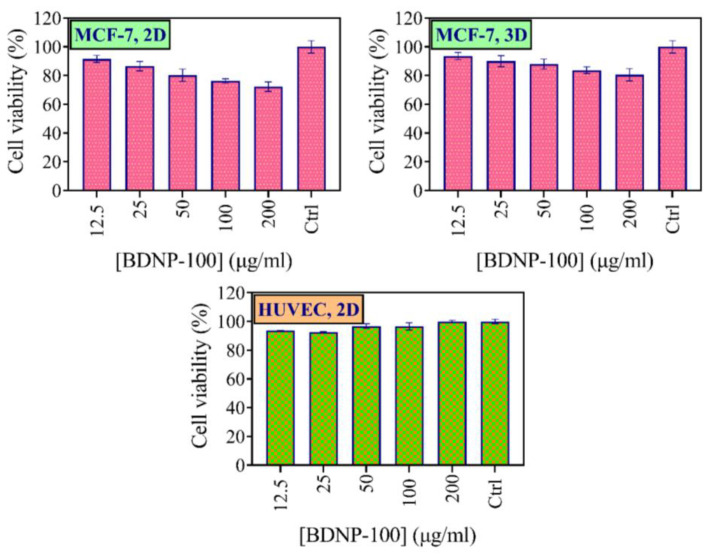
Cell viability of MCF-7 (2D, 3D) and HUVEC cells after incubation with BDNPs. All experiments were run in triplicate.

Peroxidase activity of the Hb-derived BDNPs may be an effective tool for cancer therapy. The cytotoxicity of BDNP-100 was investigated on MCF-7 as a cancer cell line, and HUVEC was investigated as a normal cell line using an MTT assay. MCF-7 cells treated with BDNP-100 demonstrated dose-dependent toxicity, and the lowest viability of around 72% was induced by 200 µg/mL of BDNP-100 (Figure 10).

We also used a three-dimensional (3D) spheroid model of MCF-7 cells for evaluating the toxicity of BDNP-100 in an in vivo-like condition. We observed dose dependency in the 3D model as well. Cell viabilities, however, were higher than the MCF-7 cells cultured in the monolayer condition (same concentration), possibly due to the higher resistance of 3D tumor models than the 2D monolayer cells to tumor treatment [58,59]. We also evaluated the cytotoxicity effect of BDNP-100 on the HUVEC cell line (Figure 10). In the same experimental conditions used for MCF-7 cells, i.e., treatment by H_2_O_2_ and BDNP-100, the viability of the HUVEC cells did not change after 24 h of incubation, indicating the ROS scavenging characteristics of the normal cells [60].

#### 3.8.2. Apoptosis

**Figure 11:**
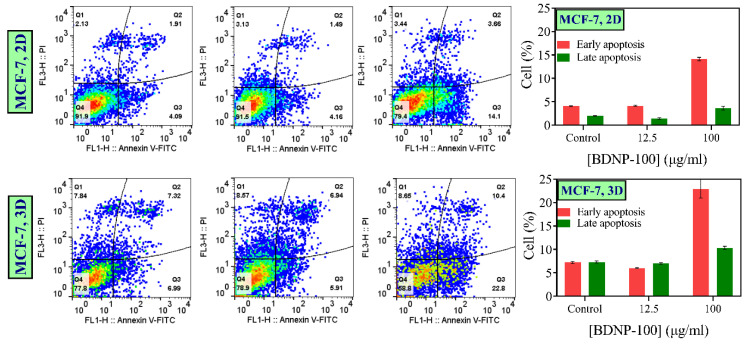
Apoptosis study of the MCF-7 cell in monolayer (2D, **upper panels**) and 3D spheroid model (**lower panels**) treated with 12.5 µg/mL and 100 µg/mL of BDNP-100.

The ability of BDNP-100 to induce apoptosis was determined by the Annexin IV-PI assay (Figure 11). The effect of low (12.5 µg/mL) and high (100 µg/mL) concentrations of BDNP-100 on the apoptosis of MCF-7 cells in the forms of monolayer and 3D spheroids was studied. The results showed that the population of cells in their early or late apoptotic stages was higher in spheroids than in monolayer cells due to the hypoxic condition of tumor-like spheroids and a lack of nutrition compounds in the spheroids’ cores. Furthermore, regardless of the culture type, the percentages of the early apoptotic cells associated with the control and the samples treated with a low concentration of BDNP-100 were very close, showing that 12.5 µg/mL of BDNP-100 induced a negligible toxic effect. In contrast, the number of early and late apoptotic cells was enhanced by increasing the concentration of BDNP-100 to 100 µg/mL. However, the rate of change in early apoptosis was much higher than the one observed for late apoptosis.

#### 3.8.3. ROS and Cell Cycle Arrest

**Figure 12:**
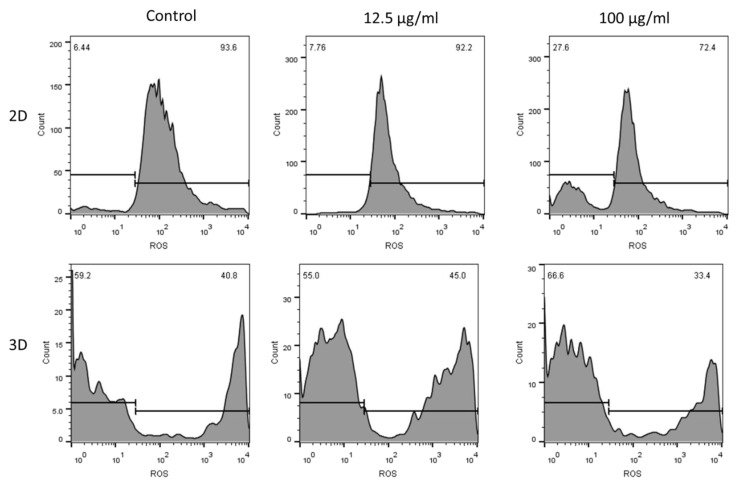
ROS levels of MCF-7 cell line as a function of BDNP concentrations after 24 h of incubation in monolayer cells and 3D spheroid models.

ROS plays an important role in redox signaling and oxidative stress [61]. Nanoparticles that are capable of ROS generation can elevate ROS beyond a certain value and harm cancer cells. In the present study, intracellular ROS levels were analyzed by flow cytometry using DCFH-DA as an oxidative probe. The fluorescence signal of the probe was measured after treatment of the MCF-7 cells with low (12.5 µg/mL) and high (100 µg/mL) concentrations of BDNP-100. Oxidative stress was measured in monolayer and 3D spheroid models. Figure 12 (upper panels) shows that ROS production at a low concentration of BDNP-100 slightly increased in the monolayer condition, indicating a low concentration of hydroxyl radicals in the cells treated with 12.5 µg/mL of BDNP-100. At 100 µg/mL, the fluorescence signal of the monolayer cells increased by around four times compared with the control.

In the 3D cellular platform (Figure 12, lower panels), a high percentage of ROS was observed in the control sample due to the inherent oxidative environment of tumor-like spheroids. A low concentration of BDNP-100 almost failed to alter the ROS level of the spheroids. The ROS level increased by around 1.1 times at a higher concentration of BDNPs (100 µg/mL). The rate of ROS production was slower in the 3D spheroids than in the monolayer condition (Figure 12). Previous reports have shown that the therapeutic efficacy of chemotherapy drugs or other modes of therapies in spheroids is lower than in monolayer cells [62]. The same trend was observed in the present study.

Flow cytometric analysis was used to look at the cell cycle profile of the MCF-7 population after different treatments (Figure S6).

The cell cycle distribution was tested to determine BDNP-triggered cell cycle arrest in MCF-7 cells grown in monolayer or spheroid cell conditions. Data for the monolayer condition showed that a low dose (12.5 µg/mL) of BDNP-100 reduced the number of cells in the G_0_/G_1_ phase from 61.7 to 56.5, and cells in their G_2_/M phase were enhanced from 15.2 to 21.4, indicating that the G_2_/M phase arrests in the cell cycle. By increasing the concentration of BDNP-100 to 100 µg/mL, the cell cycle distribution did not change compared to the control sample. Unlike the exposure in the monolayer condition, a low dose (12.5 µg/mL) of BDNP-100 in the 3D condition resulted in a marked S-phase arrest, evidenced by the high number of cells remaining in the S phase (from 17.5 to 21.9) and a reducing G_2_/M phase entry (from 15.5 to 7.05). These data suggest that BDNP-100 in the 3D condition, with high similarities to the in vivo tissues, triggered stronger growth inhibition of the MCF-7 cells than in the monolayer condition.

## 4. Conclusions

In the present study, BDNPs were synthesized from Hb molecules in biowastes. Characterization, through various techniques, demonstrated a gradual structural change in the Hb accompanied by carbonization at high treatment temperatures. Accordingly, the optical properties and peroxidase-like activity of BDNPs are strongly related to the synthesis condition. At the highest (180 °C) and lowest (100 °C) treatment temperatures, the obtained products were only fluorescent and catalytically active, respectively. Contrarily, the hydrothermal treatment of Hb at 150 °C resulted in the formation of fluorescent particles showing peroxidase-like activity. The thermal denaturation of Hb adversely affected the K_m_ value toward H_2_O_2_. However, the catalytic stability of BDNP-100 was more than that of Hb. In the optimized experimental conditions, BDNP-100 was successfully applied to obtain the quantitative measurement of H_2_O_2_. Some antioxidants interfered with H_2_O_2_-induced signal development. In combination with glucose oxidase, BDNP-100 also detected glucose with high sensitivity and selectivity. Alongside the biosensing application, BDNP-100 showed therapeutic efficacy toward cancer cells in monolayer and 3D spheroids. Flow cytometry analysis revealed that the elevated intracellular ROS in the presence of exogenous H_2_O_2_ and BDNP-100 induced cell apoptosis. The therapeutic efficacy in the 3D cellular model was less than what occurred in the monolayer conditions. The results demonstrate that catalytic particles can be prepared from biowastes through a simple hydrothermal route, showing great potential in biosensing and cancer therapy. However, a better therapeutic outcome relies on the preparation of particles with higher activity, ideally through the proposed modification of the synthesis route.

## Figures and Tables

**Table 1:** Elemental compositions of BDNP samples by XPS, EDS, and ICP-MS analyses.

		Amount		
XPS	Element	ppm	At% (BDNP-100)	At% (BDNP-150)
	O	-	12.44	15.83
	C=O	-	74.37	77.04
	C-O	-	25.63	22.96
	C	-	79.13	71.72
	C-C	-	57.95	54.53
	N-C=O	-	14.70	15.70
	C=C	-	27.35	29.77
	N	-	7.00	7.91
	C-N	-	100	94.95
	Graphitic N, N-H	-	0	5.05
	Na	-	1.44	4.54
EDS	C	-	74.55	71.61
	O	-	25.41	28.31
	Fe	-	0.05	0.07
ICP-MS	Fe (BDNP-100)	3.1	-	-
	Fe (BDNP-125)	3.3	-	-
	Fe (BDNP-150)	0.30	-	-
	Fe (BDNP-180)	0.37	-	-

**Table 2:** Michaelis–Menten kinetic parameters of different Hb and BDNP samples.

Sample	Substrate	K_m_ (mM)	V_max_ (×10^−8^, mol L^−1^ s^−1^)
Hb (blood, fresh)	H_2_O_2_	1.79	69.7
	TMB	0.18	7.1
Hb (blood, dated)	H_2_O_2_	1.54	35.5
	TMB	0.142	0.819
Hb (Sigma)	H_2_O_2_	2.00	3.64
	TMB	0.291	1.57
BDNP-100	H_2_O_2_	11.84	8.56
	TMB	0.121	0.538
BDNP-150	H_2_O_2_	11.32	3.18
	TMB	0.137	0.254
Fe-CD	H_2_O_2_	2.31	2.87
	TMB	0.134	0.789

**Table 3:** Determination of glucose in human serum and plasma samples.

Sample	Spiked (µM)	Found (µM)	Standard Method (µM)	Recovery (%)	Relative Error (%)	RSD (%, n = 3)
Sample 1 Human serum	0	76.02	77.15	-	1.46	6.60
	95	175.91	-	105.14	-	11.53
	285	385.59	-	108.62	-	1.72
	475	529.12	-	95.40	-	10.63
						
	Spiked (µM)	Found (µM)	Standard method (µM)	Recovery (%)	Relative error (%)	RSD (%, n = 3)
Sample 2 Human plasma	0	87.38	80.38	-	8.71	14.8
	95	192.65	-	110.81	-	1.53
	285	378.16	-	102.03	-	5.34
	475	549.20	-	97.22	-	7.60
						
	Spiked (µM)	Found (µM)	Standard method (µM)	Recovery (%)	Relative error (%)	RSD (%, n = 3)
Sample 3 Human serum	0	102.69	109.00	-	5.78	2.80
	285	392.44	-	101.67	-	5.96
	475	571.59	-	98.72	-	4.13
						
	Spiked (µM)	Found (µM)	Standard method (µM)	Recovery (%)	Relative error (%)	RSD (%, n = 3)
Sample 4 Human serum	0	93.12	86.81	-	7.27	1.76
	285	373.82	-	98.49	-	8.94
	475	555.93	-	97.43	-	13.44

## Data Availability

The data presented in this study are available on request from the corresponding author. The XPS and EDS mapping of BDNP-100 and BDNP-150, spectral data of hemoglobin and BDNP samples, and cell cycle experiments are provided in Supplementary Materials.

## Supplementary Materials

The following supporting information can be downloaded at: https://www.mdpi.com/article/10.3390/bios13060583/s1, Figure S1: (A) XPS survey spectrum and high-resolution (B) C1s, (C) N1s, and (D) O1s spectra of BDNP-100. (E) Sulfur region. Figure S2: (A) XPS survey spectrum and high-resolution (B) C1s, (C) N1s, and (D) O1s spectra of BDNP-150. (E) Sulfur region. Figure S3: EDS elemental mapping of BDNP-100 and BDNP-150. Figure S4: (A) UV–Vis and (B) fluorescence spectra of BDNP samples under excitation wavelengths of 250 nm and 300 nm. Figure S5: UV–Vis absorption spectra of Hb samples of two origins dispersed in deionized water. Figure S6: Flow cytometric analysis of the cell cycle of MCF-7 cell lines treated with BDNPs at concentrations of 12.5 and 100 μg/mL in monolayer and 3D spheroid model conditions; Table S1: Performance of glucose colorimetric sensors. References in Supplementary Materials are [30,31,32,33,35,37,63,64,65,66,67,68,69,70,71,72,73].

## References

[1] Mohammadpour-Haratbar A., Zare Y., Rhee K.Y. (2022). Electrochemical biosensors based on polymer nanocomposites for detecting breast cancer: Recent progress and future prospects. Adv. Colloid Interface Sci..

[2] Boraei S.B.A., Nourmohammadi J., Mahdavi F.S., Zare Y., Rhee K.Y., Montero A.F., Herencia A.J.S., Ferrari B. (2022). Osteogenesis capability of three-dimensionally printed poly (lactic acid)-halloysite nanotube scaffolds containing strontium ranelate. Nanotechnol. Rev..

[3] Mohammadpour-Haratbar A., Boraei S.B.A., Zare Y., Rhee K.Y., Park S.-J. (2023). Graphene-Based Electrochemical Biosensors for Breast Cancer Detection. Biosensors.

[4] Zare Y., Rhee K.Y. (2022). Effect of contact resistance on the electrical conductivity of polymer graphene nanocomposites to optimize the biosensors detecting breast cancer cells. Sci. Rep..

[5] Mohammadpour-Haratbar A., Mohammadpour-Haratbar S., Zare Y., Rhee K.Y., Park S.-J. (2022). A Review on Non-Enzymatic Electrochemical Biosensors of Glucose Using Carbon Nanofiber Nanocomposites. Biosensors.

[6] Sadrabadi E.A., Khosravi F., Benvidi A., Dezfuli A.S., Khashayar P., Khashayar P., Azimzadeh M. (2022). Alprazolam Detection Using an Electrochemical Nanobiosensor Based on AuNUs/Fe-Ni@ rGO Nanocomposite. Biosensors.

[7] Gao L., Zhuang J., Nie L., Zhang J., Zhang Y., Gu N., Wang T., Feng J., Yang D., Perrett S. (2007). Intrinsic peroxidase-like activity of ferromagnetic nanoparticles. Nat. Nanotechnol..

[8] Jiang D., Ni D., Rosenkrans Z.T., Huang P., Yan X., Cai W. (2019). Nanozyme: New horizons for responsive biomedical applications. Chem. Soc. Rev..

[9] Wang H., Wan K., Shi X. (2019). Recent Advances in Nanozyme Research. Adv. Mater..

[10] Cai X., Jiao L., Yan H., Wu Y., Gu W., Du D., Lin Y., Zhu C. (2021). Nanozyme-involved biomimetic cascade catalysis for biomedical applications. Mater. Today.

[11] Huang Y., Ren J., Qu X. (2019). Nanozymes: Classification, catalytic mechanisms, activity regulation, and applications. Chem. Rev..

[12] Wang P., Wang T., Hong J., Yan X., Liang M. (2020). Nanozymes: A New Disease Imaging Strategy. Front. Bioeng. Biotechnol..

[13] Wu J., Wang X., Wang Q., Lou Z., Li S., Zhu Y., Qin L., Wei H. (2019). Nanomaterials with enzyme-like characteristics (nanozymes): Next-generation artificial enzymes (II). Chem. Soc. Rev..

[14] Hendrickson O.D., Zvereva E.A., Panferov V.G., Solopova O.N., Zherdev A.V., Sveshnikov P.G., Dzantiev B.B. (2022). Application of Au@ Pt Nanozyme as Enhancing Label for the Sensitive Lateral Flow Immunoassay of Okadaic Acid. Biosensors.

[15] Zhou L., Liu Y., Lu Y., Zhou P., Lu L., Lv H., Hai X. (2022). Recent Advances in the Immunoassays Based on Nanozymes. Biosensors.

[16] Vu T.H., Nguyen P.T., Kim M.I. (2022). Polydopamine-Coated Co3O4 Nanoparticles as an Efficient Catalase Mimic for Fluorescent Detection of Sulfide Ion. Biosensors.

[17] Siddiquee M.A., Parray M.U.D., Kamli M.R., Malik M.A., Mehdi S.H., Imtiyaz K., Rizvi M.M.A., Rajor H.K., Patel R. (2021). Biogenic synthesis, in-vitro cytotoxicity, esterase activity and interaction studies of copper oxide nanoparticles with lysozyme. J. Mater. Res. Technol..

[18] Smutok O., Kavetskyy T., Prokopiv T., Serkiz R., Šauša O., Novák I., Švajdlenková H., Maťko I., Gonchar M., Katz E. (2022). Biosensor Based on Peroxidase-Mimetic Nanozyme and Lactate Oxidase for Accurate L-Lactate Analysis in Beverages. Biosensors.

[19] Chen Y., Gao X., Xue H., Liu G., Zhou Y., Peng J. (2022). One-Pot Preparation of Imidazole-Ring-Modified Graphitic Carbon Nitride Nanozymes for Colorimetric Glucose Detection. Biosensors.

[20] Liang M., Fan K., Pan Y., Jiang H., Wang F., Yang D., Lu D., Feng J., Zhao J., Yang L. (2013). Fe_3_O_4_ Magnetic Nanoparticle Peroxidase Mimetic-Based Colorimetric Assay for the Rapid Detection of Organophosphorus Pesticide and Nerve Agent. Anal. Chem..

[21] Lu C., Liu X., Li Y., Yu F., Tang L., Hu Y., Ying Y. (2015). Multifunctional Janus Hematite–Silica Nanoparticles: Mimicking Peroxidase-Like Activity and Sensitive Colorimetric Detection of Glucose. ACS Appl. Mater. Interfaces.

[22] Zhang T., Cao C., Tang X., Cai Y., Yang C., Pan Y. (2016). Enhanced peroxidase activity and tumour tissue visualization by cobalt-doped magnetoferritin nanoparticles. Nanotechnology.

[23] Ni P., Sun Y., Dai H., Lu W., Jiang S., Wang Y., Li Z., Li Z. (2017). Prussian blue nanocubes peroxidase mimetic-based colorimetric assay for screening acetylcholinesterase activity and its inhibitor. Sens. Actuators B Chem..

[24] Shi W., Wang Q., Long Y., Cheng Z., Chen S., Zheng H., Huang Y. (2011). Carbon nanodots as peroxidase mimetics and their applications to glucose detection. Chem. Commun..

[25] Shamsipur M., Safavi A., Mohammadpour Z. (2014). Indirect colorimetric detection of glutathione based on its radical restoration ability using carbon nanodots as nanozymes. Sens. Actuators B Chem..

[26] Mohammadpour Z., Safavi A., Shamsipur M. (2014). A new label free colorimetric chemosensor for detection of mercury ion with tunable dynamic range using carbon nanodots as enzyme mimics. Chem. Eng. J..

[27] Wang J., Huang R., Qi W., Su R., Binks B.P., He Z. (2019). Construction of a bioinspired laccase-mimicking nanozyme for the degradation and detection of phenolic pollutants. Appl. Catal. B Environ..

[28] Mohammadpour Z., Hashemi Z.S., Malekian Jebeli F., Ghasemzadeh S., Askari E., Akbary-Yekta M., Sarrami-Forooshani R. (2021). Iron Oxychloride/Bovine Serum Albumin Nanosheets as Chemodynamic Therapy Agents. Part. Part. Syst. Charact..

[29] Tang G., He J., Liu J., Yan X., Fan K. (2021). Nanozyme for tumor therapy: Surface modification matters. Exploration.

[30] Yin X., Liu P., Xu X., Pan J., Li X., Niu X. (2021). Breaking the pH limitation of peroxidase-like CoFe2O4 nanozyme via vitriolization for one-step glucose detection at physiological pH. Sens. Actuators B Chem..

[31] Zhao W., Zhang G., Du Y., Chen S., Fu Y., Xu F., Xiao X., Jiang W., Ji Q. (2021). Sensitive colorimetric glucose sensor by iron-based nanozymes with controllable Fe valence. J. Mater. Chem. B.

[32] Wahab M.A., Hossain S.M.A., Masud M.K., Park H., Ashok A., Mustapić M., Kim M., Patel D., Shahbazi M., Hossain M.S.A. (2022). Nanoarchitectured superparamagnetic iron oxide-doped mesoporous carbon nanozymes for glucose sensing. Sens. Actuators B Chem..

[33] Feng L., Zhang L., Chu S., Zhang S., Chen X., Du Z., Gong Y., Wang H. (2022). Controllable doping of Fe atoms into MoS2 nanosheets towards peroxidase-like nanozyme with enhanced catalysis for colorimetric analysis of glucose. Appl. Surf. Sci..

[34] Zha J., Wu W., Xie P., Han H., Fang Z., Chen Y., Jia Z. (2022). Polymeric Nanocapsule Enhances the Peroxidase-like Activity of Fe_3_O_4_ Nanozyme for Removing Organic Dyes. Catalysts.

[35] Wang Q., Zhang X., Huang L., Zhang Z., Dong S. (2017). One-Pot Synthesis of Fe_3_O_4_ Nanoparticle Loaded 3D Porous Graphene Nanocomposites with Enhanced Nanozyme Activity for Glucose Detection. ACS Appl. Mater. Interfaces.

[36] Bao Y., Shi C., Wang T., Li X., Ma J. (2016). Recent progress in hollow silica: Template synthesis, morphologies and applications. Microporous Mesoporous Mater..

[37] Dong W., Chen G., Hu X., Zhang X., Shi W., Fu Z. (2020). Molybdenum disulfides nanoflowers anchoring iron-based metal organic framework: A synergetic catalyst with superior peroxidase-mimicking activity for biosensing. Sens. Actuators B Chem..

[38] Mu Z., Wu S., Guo J., Zhao M., Wang Y. (2022). Dual Mechanism Enhanced Peroxidase-like Activity of Iron–Nickel Bimetal–Organic Framework Nanozyme and Its Application for Biosensing. ACS Sustain. Chem. Eng..

[39] Chen M., Zhou H., Liu X., Yuan T., Wang W., Zhao C., Zhao Y., Zhou F., Wang X., Xue Z. (2020). Single Iron Site Nanozyme for Ultrasensitive Glucose Detection. Small.

[40] Bandi R., Alle M., Park C.-W., Han S.-Y., Kwon G.-J., Kim N.-H., Kim J.-C., Lee S.-H. (2021). Cellulose nanofibrils/carbon dots composite nanopapers for the smartphone-based colorimetric detection of hydrogen peroxide and glucose. Sens. Actuators B Chem..

[41] Zhang Z., Hao J., Zhang J., Zhang B., Tang J. (2012). Protein as the source for synthesizing fluorescent carbon dots by a one-pot hydrothermal route. RSC Adv..

[42] Tan M., Li X., Wu H., Wang B., Wu J. (2015). N-doped carbon dots derived from bovine serum albumin and formic acid with one- and two-photon fluorescence for live cell nuclear imaging. Colloids Surf. B Biointerfaces.

[43] Liu X., Li T., Hou Y., Wu Q., Yi J., Zhang G. (2016). Microwave synthesis of carbon dots with multi-response using denatured proteins as carbon source. RSC Adv..

[44] Chowdhury Z.Z., Krishnan B., Sagadevan S., Rafique R.F., Hamizi N.A.B., Abdul Wahab Y., Khan A.A., Johan R.B., Al-Douri Y., Kazi S.N. (2018). Effect of Temperature on the Physical, Electro-Chemical and Adsorption Properties of Carbon Micro-Spheres Using Hydrothermal Carbonization Process. Nanomaterials.

[45] Jiang Y., Li C., Nguyen X., Muzammil S., Towers E., Gabrielson J., Narhi L. (2011). Qualification of FTIR spectroscopic method for protein secondary structural analysis. J. Pharm. Sci..

[46] Kong J., Yu S. (2007). Fourier Transform Infrared Spectroscopic Analysis of Protein Secondary Structures. Acta Biochim. Biophys. Sin..

[47] Mallamace F., Baglioni P., Corsaro C., Chen S.-H., Mallamace D., Vasi C., Stanley H.E. (2014). The influence of water on protein properties. J. Chem. Phys..

[48] Zhou L., Yang Y., Ren H., Zhao Y., Wang Z., Wu F., Xiao Z. (2016). Structural Changes in Rice Bran Protein upon Different Extrusion Temperatures: A Raman Spectroscopy Study. J. Chem..

[49] Tan C., Zuo S., Zhao Y., Shen B. (2019). Preparation of multicolored carbon quantum dots using HNO_3_/HClO_4_ oxidation of graphitized carbon. J. Mater. Res..

[50] Wang L., Hu N. (2001). Direct electrochemistry of hemoglobin in layer-by-layer films with poly(vinyl sulfonate) grown on pyrolytic graphite electrodes. Bioelectrochemistry.

[51] Paynter R.W., Ratner B.D., Horbett T.A., Thomas H.R. (1984). XPS studies on the organization of adsorbed protein films on fluoropolymers. J. Colloid Interface Sci..

[52] Ogi T., Aishima K., Permatasari F.A., Iskandar F., Tanabe E., Okuyama K. (2016). Kinetics of nitrogen-doped carbon dot formation via hydrothermal synthesis. New J. Chem..

[53] Miao X., Yan X., Qu D., Li D., Tao F.F., Sun Z. (2017). Red Emissive Sulfur, Nitrogen Codoped Carbon Dots and Their Application in Ion Detection and Theraonostics. ACS Appl. Mater. Interfaces.

[54] Greenstein J.P. (1938). Sulfhydryl groups in proteins: I. egg albumin in solutions of urea, guanidine, and their derivatives. J. Biol. Chem..

[55] Meng F., Alayash A.I. (2017). Determination of extinction coefficients of human hemoglobin in various redox states. Anal. Biochem..

[56] Zheng N., Lian Y., Zhou Q., Wang R., He X., Hu R., Hu Z. (2022). An effective Fenton reaction by using waste ferriciron and red phosphorus. Chem. Eng. J..

[57] Buettner G.R., Jurkiewicz B.A. (1996). Catalytic Metals, Ascorbate and Free Radicals: Combinations to Avoid. Radiat. Res..

[58] Hakanson M., Kobel S., Lutolf M.P., Textor M., Cukierman E., Charnley M. (2012). Controlled breast cancer microarrays for the deconvolution of cellular multilayering and density effects upon drug responses. PLoS ONE.

[59] Gong X., Lin C., Cheng J., Su J., Zhao H., Liu T., Wen X., Zhao P. (2015). Generation of Multicellular Tumor Spheroids with Microwell-Based Agarose Scaffolds for Drug Testing. PLoS ONE.

[60] Guo J., Wang Y., Zhao M. (2019). Target-directed functionalized ferrous phosphate-carbon dots fluorescent nanostructures as peroxidase mimetics for cancer cell detection and ROS-mediated therapy. Sens. Actuators B Chem..

[61] Schieber M., Chandel N.S. (2014). ROS function in redox signaling and oxidative stress. Curr. Biol. CB.

[62] Jamali T., Kavoosi G., Safavi M., Ardestani S.K. (2018). In-vitro evaluation of apoptotic effect of OEO and thymol in 2D and 3D cell cultures and the study of their interaction mode with DNA. Sci. Rep..

[63] Guidet B., Shah S.V. (1989). Enhanced in vivo H_2_O_2_ generation by rat kidney in glycerol-induced renal failure. Am. J. Physiol..

[64] Jiang Z.Y., Woollard A.C., Wolff S.P. (1990). Hydrogen peroxide production during experimental protein glycation. FEBS Lett..

[65] Thannickal V.J., Fanburg B.L. (1995). Activation of an H_2_O_2_-generating NADH oxidase in human lung fibroblasts by transforming growth factor beta 1. J. Biol. Chem..

[66] Andrade C.T., Barros L.A.M., Lima M.C.P., Azero E.G. (2004). Purification and characterization of human hemoglobin: Effect of the hemolysis conditions. Int. J. Biol. Macromol..

[67] Schneider J., Reckmeier C.J., Xiong Y., von Seckendorff M., Susha A.S., Kasák P., Rogach A.L. (2017). Molecular Fluorescence in Citric Acid-Based Carbon Dots. J. Phys. Chem. C.

[68] Chen Y., Yuchi Q., Li T., Yang G., Miao J., Huang C., Liu J., Li A., Qin Y., Zhang L. (2020). Precise engineering of ultra-thin Fe_2_O_3_ decorated Pt-based nanozymes via atomic layer deposition to switch off undesired activity for enhanced sensing performance. Sens. Actuators B Chem..

[69] Zhao Z., Huang Y., Liu W., Ye F., Zhao S. (2020). Immobilized Glucose Oxidase on Boronic Acid-Functionalized Hierarchically Porous MOF as an Integrated Nanozyme for One-Step Glucose Detection. ACS Sustain. Chem. Eng..

[70] Xian J., Weng Y., Guo H., Li Y., Yao B., Weng W. (2019). One-pot fabrication of Fe-doped carbon nitride nanoparticles as peroxidase mimetics for H_2_O_2_ and glucose detection. Spectrochim. Acta Part A Mol. Biomol. Spectrosc..

[71] Zhong Y., Yang J., Yin X., Zheng J., Lu N., Zhang M. (2019). Enhanced synergistic effects from multiple iron oxide nanoparticles encapsulated within nitrogen-doped carbon nanocages for simple and label-free visual detection of blood glucose. Nanotechnology.

[72] Chen H., Yuan C., Yang X., Cheng X., Elzatahry A.A., Alghamdi A., Su J., He X., Deng Y. (2020). Hollow Mesoporous Carbon Nanospheres Loaded with Pt Nanoparticles for Colorimetric Detection of Ascorbic Acid and Glucose. ACS Appl. Nano Mater..

[73] Li T., Hu P., Li J., Huang P., Tong W., Gao C. (2019). Enhanced peroxidase-like activity of Fe@PCN-224 nanoparticles and their applications for detection of H_2_O_2_ and glucose. Colloids Surf. A Physicochem. Eng. Asp..

