# Supplementary material for: Synthesis of Fe-Doped Peroxidase Mimetic Nanozymes from Natural Hemoglobin for Colorimetric Biosensing and In Vitro Anticancer Effects
Source: Biosensors (Basel). 2023 May 27;13(6):583. doi: 10.3390/bios13060583 (PMC10296309; doi:10.3390/bios13060583)
Supplement: Supplementary file 1 [file biosensors-13-00583-s001.zip › biosensors-2381954-supplementary.pdf]

biosensors

MDPI

Supplementary Materials

Synthesis of Fe-Doped Peroxidase Mimetic Nanozymes from Natural Hemoglobin for Colorimetric Biosensing and In Vitro Anticancer Effects

Zahra Mohammadpour [1,*], Esfandyar Askari [1], Farhad Shokati [1], Hosna Sadat Hoseini [1], Mojtaba Kamankesh [1], Yasser Zare [1] and Kyong Yop Rhee [2,*]

[1] Biomaterials and Tissue Engineering Department, Breast Cancer Research Center, Motamed Cancer Institute, ACECR, Tehran 1517964311, Iran; esfandyaraskari@uvic.ca (E.A.); shokati@acecr.ac.ir (F.S.); hoseini.h.s@ut.ac.ir (H.S.H.); m.kamankesh@ut.ac.ir (M.K.); y.zare@aut.ac.ir (Y.Z.)

[2] Department of Mechanical Engineering (BK21 Four), College of Engineering, Kyung Hee University, Yongin 17104, Republic of Korea

* Correspondence: mohammadpour@acecr.ac.ir (Z.M.); rheeky@khu.ac.kr (K.Y.R.)

Materials and Methods:

Instruments

The elemental composition of BDNPs was identified by Energy-dispersive X-ray spectroscopy (EDS) analysis (MIRA3TESCAN-XMU). A FEI Tecnai G2 F20 SuperTwin (accelerating voltage: 200 kV) transmission electron microscope (TEM) was used to evaluate the morphology of the BDNPs. An ELx800 ™ microplate absorbance reader (BioTek Instruments) and a UV-Vis spectrophotometer (Biowave II) were used for acquiring the UV-Vis spectroscopic data. Synthesis of blood-derived BDNPs was accomplished in an AZAR furnace with controllable temperature. Hemolysis of red blood cells (RBCs) was done using a Qsonica probe sonicator (Q700). The Fourier transform infrared (FTIR) spectra were measured with a PerkinElmer Spectrum 65 spectrometer over the spectral range of 450–4000 cm^{-1}. Fluorescence measurements were carried out by a RF-6000 spectrofluorometer. The Fe content of BDNPs was measured by ICP-MS (Perkin Elmer). The H_2O_2 concentration of the stock solution was determined based on its extinction coefficient of 43.6 M^{-1} cm^{-1} at 240 nm [1-3].

Extraction of Hb from human whole blood

5 mL of a blood biowaste from healthy volunteer was obtained from the Iranian Blood Transfusion Organization. The RBCs were isolated by gradient centrifugation using Ficoll-Hypaque 1077 (GE health care-Sweden). Hb extraction was performed according to a reported procedure[4]. Briefly, the isolated RBCs were washed with an equal weight of isotonic saline solution (0.9% NaCl, w/v) and centrifuged at 1000*g* for 30 min to remove the remaining human serum plasma. The supernatant was discarded. The RBCs were redispersed in 0.01M NaCl, considering a 1:2 (w/w) ratio of RBC/NaCl solution. Hemolysis was carried out with a probe sonicator equipped with a 13 mm diameter probe (amplitude 20%,10 min, pulse-on time 1s, pulse-off time 2s). After breaking down the RBCs by ultrasonication, the suspension of lysed cells was heated at 60 °C for 1 h in a water bath in the dark, and centrifuged at 2000g for 1 h. The precipitate was discarded and the clear dark red supernatant was used for the synthesis experiments.

Cell culture

Human breast cancer cell line (MCF-7) and human umbilical vein endothelial cells (HUVEC) were provided by the National Cell Bank of Iran and cultivated in Dulbecco's Modified Eagle Medium (DMEM high-glucose) with 10% FBS and 1% penicillin-streptomycin. The cells were incubated at 37°C with 5% CO_2. The cells were then re- suspended with 0.25% Trypsin-EDTA and tests were performed on cells at 80% confluence between passages 3 and 6.

3D cell spheroid formation

For the spheroid formation and 3D studies, we selected MCF-7 cells to mimic a microtumor environment. Extremely high density in a hanging drop was favored for the production of spheroids and to provide the cells with an in vivo milieu. The cells were cultured in the form of 20 µL drops on the inverted lid of a 35 mm petri dish with Ca^{+2}- and Mg^{+2}-free HBSS buffer supplemented with streptomycin (100 µg/mL), gentamycin (50 µg/mL), and penicillin 100 (U/mL).

MTT assay

MCF-7 and HUVEC cell lines were seeded into 96 well plates (9000 cells per well) and incubated overnight in the monolayer cell culture environment. In the culture media, a stock BDNP-100 stock solution (1mg/mL) was prepared, and diluted solutions were replaced with culture medium. Control wells only contained culture media. Cell proliferation and viability were evaluated using the MTT test to investigate the cytotoxic effects of BDNP-100 on MCF-7 and HUVEC cells. After 24 hours of treatment, the BDNP-containing culture media

was withdrawn, and the wells were treated with 50 µM H_2O_2 for 6 hours. The cells were then subjected to 50µL MTT reagent (0.5 mg/mL) for 3 hours at 37 °C. The insoluble formazan crystals were then dissolved in 150 µL DMSO. After 30 minutes, absorbance values at 570 and 630 nm were measured using a microplate reader (ELx800 ™, Biotech, Winooski, VT).

Apoptosis test

MCF-7 cells (in monolayer and cell spheroid conditions) and controls were seeded separately into 6cm plates at a density of 2×10^5 cells/dish. 12 hours after adhesion, cells were treated with low (12.5 µg/mL) and high (100 µg/mL) doses of BDNP-100 for 24 hours. In the following, cells were treated with H_2O_2 (50 µM) for 6 hours. To identify apoptotic cells, an Annexin VFITC/propidium iodide (PI) apoptosis test was employed. Cells were trypsinized, washed twice with PBS, and suspended in 100 µL of 1X Annexin V binding solution. Following that, 5 µL of Annexin V-FITC and PI staining solutions were added to the cell suspension, which was then incubated in the dark at room temperature for 15 minutes. Apoptosis was identified using flow cytometry (BD FACS Calibur (BD biosciences, San Jose, CA, USA)) and analyzed with FlowJo after the addition of 400 µL 1X Annexin V binding solution. The apoptotic cell percentage was measured as Annexin V-positive cells/total cell number.

ROS assay

Flow cytometry was used to detect intracellular ROS inside the cancer cells. MCF-7 cells (in monolayer and cell spheroid condition) and their controls were treated with low (12.5 µg/mL) and high (100 µg/mL) doses of BDNP-100 for 24 hours. In the following, cells were treated with H_2O_2 (50 µM) for 6 hours. MCF-7 cells were collected and treated with 10 µM 2'7'dichlorofluorescin diacetate (DCFH-DA) dye for 20 minutes at 37 °C. Flow cytometry was used to assess the fluorescence intensity of each sample and FlowJo software was used to analyze the data.

Cell Cycle

MCF-7 cells (monolayer and cell spheroids) and their controls were treated with low (12.5 µg/mL) and high (100 µg/mL) doses of BDNP-100 for 24 hours. In the following, cells were treated with H_2O_2 (50 µM) for 6 hours. Then, cells were collected and fixed overnight at 4 °C in 70% ethanol. After removing the fixative solution by centrifugation at 200g for 5 minutes at room temperature, cell pellets were washed twice with ice-cold PBS and treated with RNase A (10 g/mL; cat. no. ST579; Beyotime Institute of Biotechnology) and PI (final concentration, 50 g/mL) for 30 minutes at room temperature in the dark. PI-stained cells were analyzed for cell cycle phase distribution using flow cytometry within 1 hour. FlowJo v10 was used to examine cell phase dispersion.

UV-Vis and PL of BDNP samples:

The spectral features of the extracted Hb coincide well with the spectral pattern of pure Hb in the literature (**Fig. S4**). The UV-Vis absorption spectrum of BDNPs obtained from hydrothermally-treated Hb at 100 °C (BDNP-100), shows a sharp absorption peak at around 400 nm, which is consistent with the Soret band of native Hb. Except that, none of the peaks that originally exist in the UV-Vis absorption spectrum of Hb are seen in the UV-Vis spectrum of BDNP-100.

It is possible that chemical transformation of Hb upon thermal treatment of the sample at 100 °C has proceeded partially. By increasing the synthesis temperature to 125 °C and 150 °C, the peak intensity at 400 nm decreases and disappears for BDNP-180. The spectral profile of the particles synthesized at 180 °C is close to the commonly observed absorption spectrum of carbon nanodots [5].

Under the UV light excitation at 365 nm, we observed that samples prepared at 150 °C (BDNP-150) and 180 °C (BDNP-180) glow while the other two (BDNP-100 and BDNP-125) did not exhibit any fluorescence signal that could be detected by the naked eye. To evaluate and compare the emission profiles, all samples were excited by two wavelengths (250 nm and 300 nm), and the corresponding emission spectra were recorded by a spectrofluorometer. As seen in **Fig. S4b, c**, native Hb exhibits one emission peak at 300-350 nm and another weaker peak at around 450 nm. Regardless of the excitation wavelength, the emission profiles of BDNP-100 and BDNP-125 are similar to Hb. In contrast, BDNP-150 and BDNP-180 emit a blue light upon UV excitation (**Fig. S4a**, inset). Accordingly, a single emission peak at 400-420 nm is observed for BDNP-150, BDNP-180, similar to the emission profile of carbon nanodots in the previous reports. Based on the UV-Vis and fluorescence emission data, we inspect that at low temperatures (100 and 125 °C), chemical transformation of Hb is incomplete, while samples treated at 150 and 180 °C carbonize into fluorescent particles.

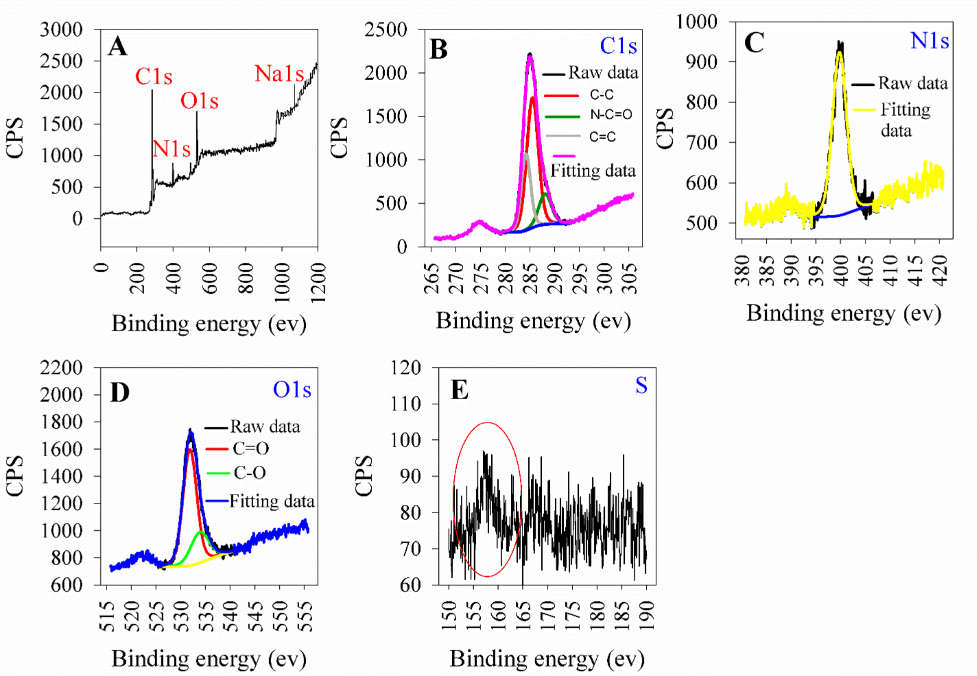

Figure S1. (A) XPS survey spectrum and high resolution (B) C1s, (C) N1s, and (D) O1s, spectra of BDNP-100. (E) sulfur region.

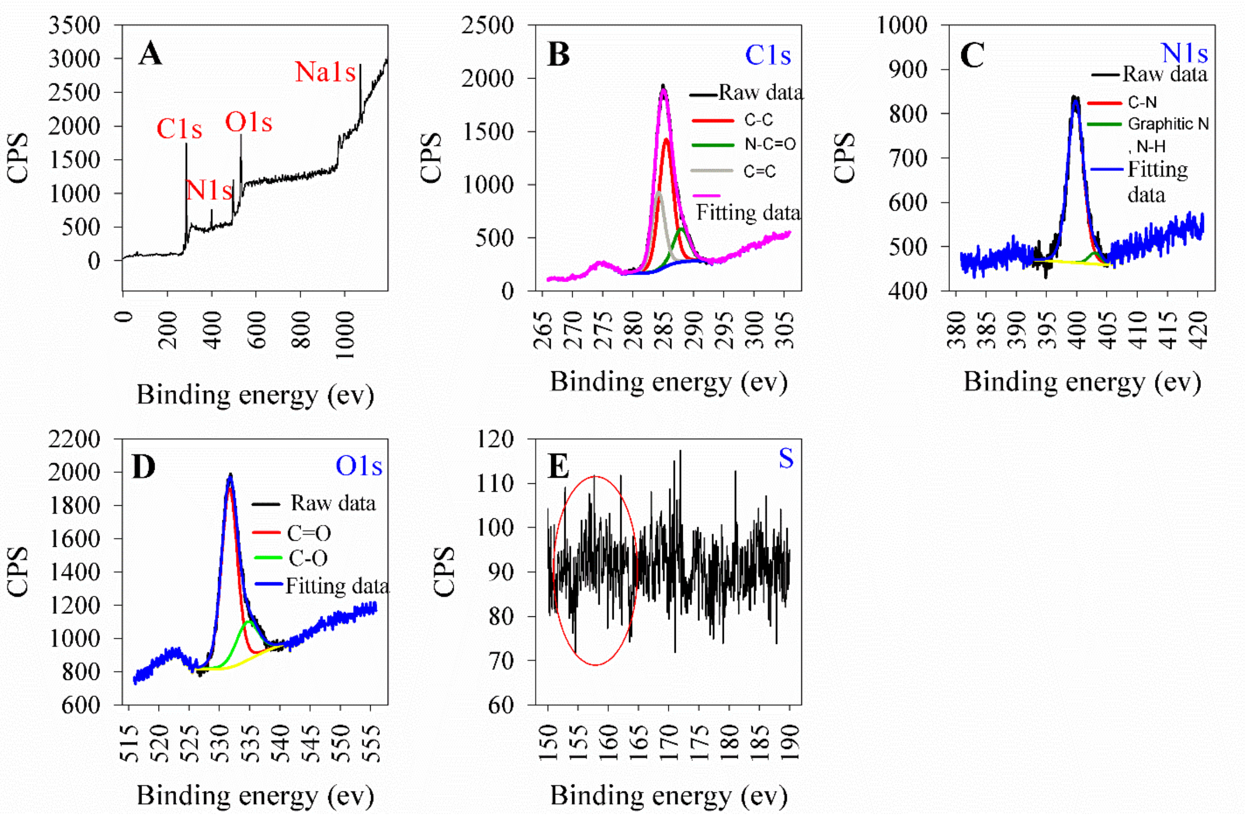

Figure S2. (A) XPS survey spectrum and high resolution (B) C1s, (C) N1s, and (D) O1s, spectra of BDNP-150. (E) sulfur region.

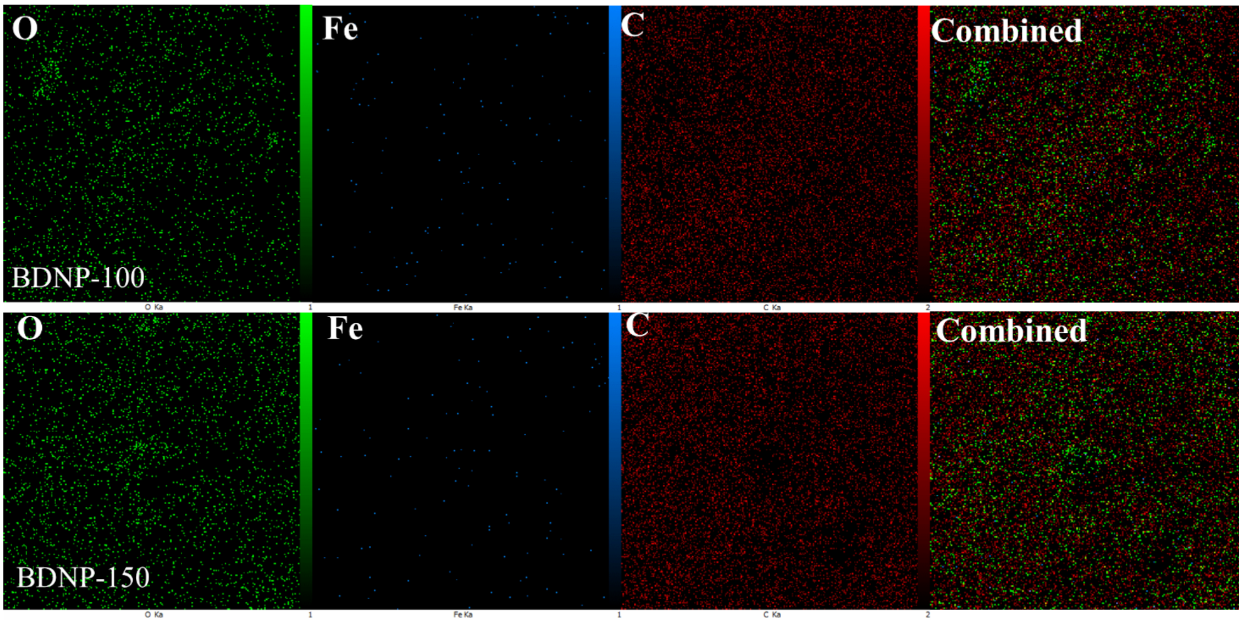

Figure S3. EDS elemental mapping of BDNP-100 and BDNP-150.

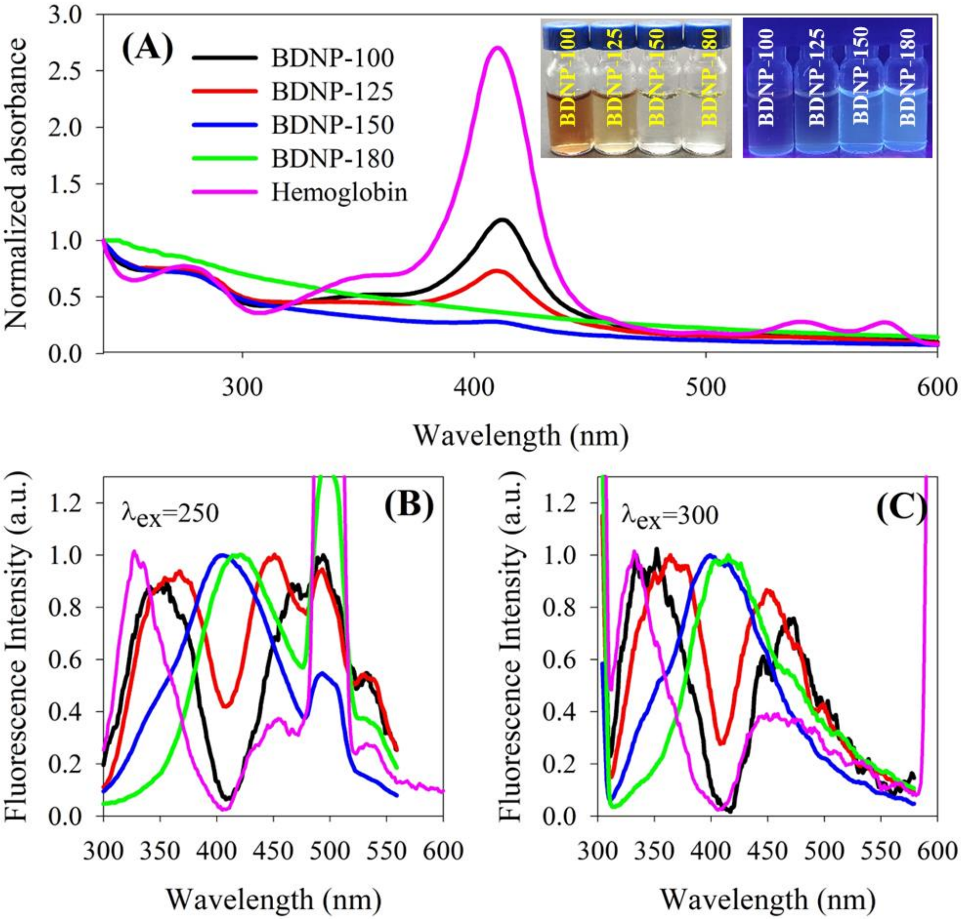

Figure S4. (A) the UV-Vis and (B) fluorescence spectra of BDNP samples under the excitation wavelengths of 250 nm and 300 nm.

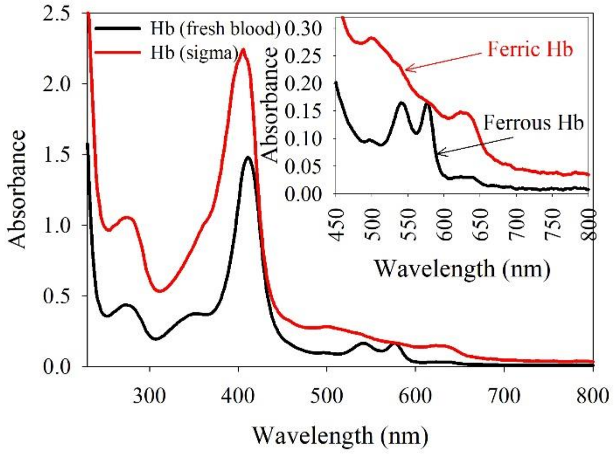

Figure S5. UV-Vis absorption spectra of Hb samples of two origins dispersed in deionized water.

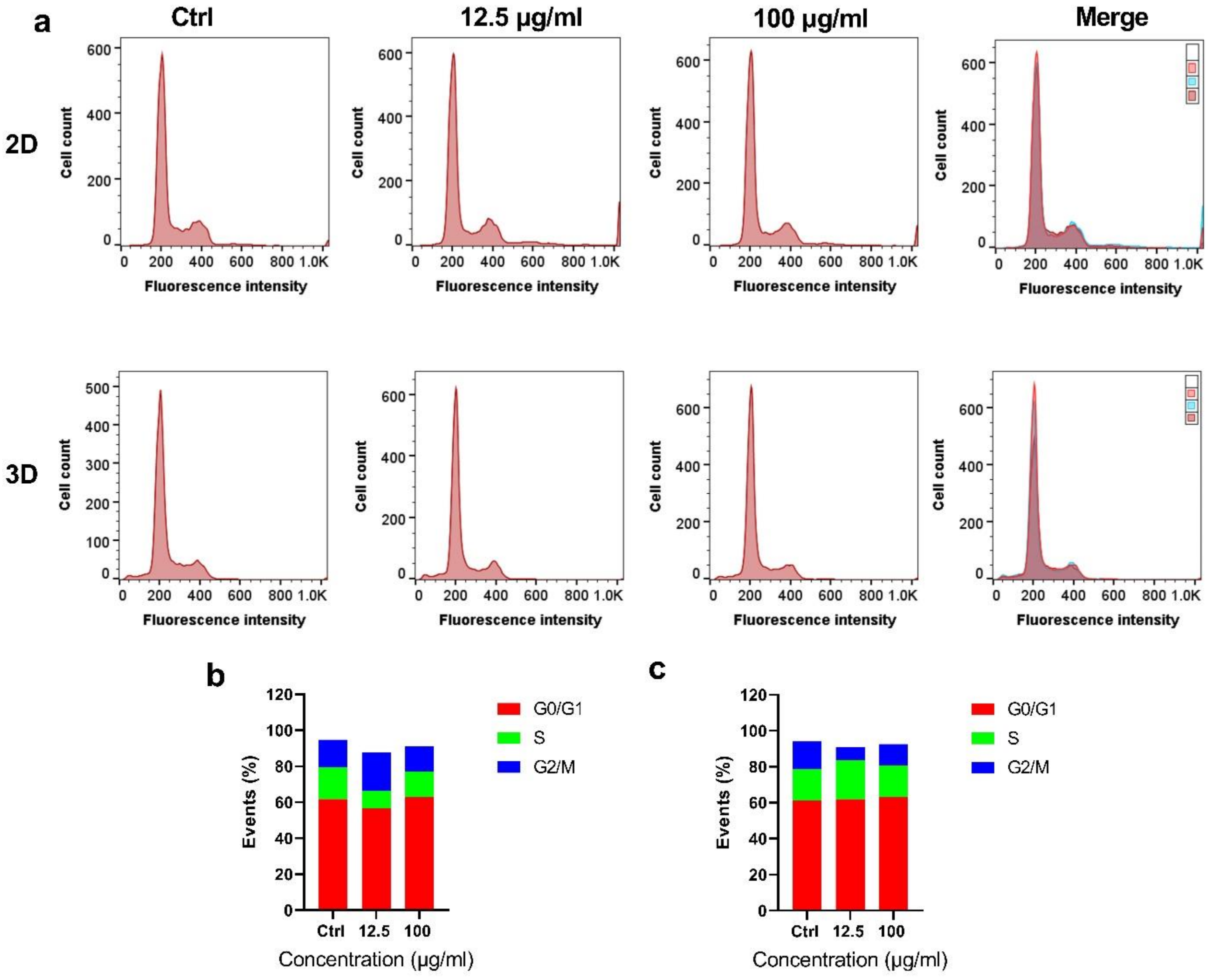

Figure S6. Flow cytometric analysis of the cell cycle of MCF-7 cell lines treated with BDNPs at concentrations of 12.5 and 100 µg/mL in monolayer and 3D spheroid model condition.

Table S1. Performance of glucose colorimetric sensors.

	**Linear range (μM)**	**LoD (μM)**	**Response time (min)**	**Reference**
single iron site nanozyme on porous N-doped carbon	10-60	2.1	10	[6]
MoS_2-MIL-101(Fe)	0.01-20	0.01	20	[7]
$2Fe_2O_3$/30Pt/CNTs	5-20	0.92	-	[8]
GOx@HP-MIL88B-BA	2-100	0.98	10	[9]
Fe-CNNPs	0-100	0.29	10	[10]
FNHCs	1.25-25	1.7	10	[11]
$SO_4^{2-}/CoFe_2O_4$	0-300	6.4	10	[12]
Pt nanoparticles/ hollow mesoporous carbon nanospheres	100–1330	35.4	30	[13]
Fe doped silica hollow spheres	5-600	0.24	5	[14]
3D porous graphene decorated with Fe_3O_4 NPs	5-500	0.8	10 [a]	[15]
Fe@PCN-224	30-800	22	10	[16]
superparamagnetic iron oxide-doped mesoporous carbon	25-10000 (R2=0.9415)	2	10	[17]
Fe-MoS_2	5-2000	1.2	-	[18]
BDNP	50-700	40	4	This work

[a] measurement was made at 50 °C

References

[1] B. Guidet, S.V. Shah, Enhanced in vivo H2O2 generation by rat kidney in glycerol-induced renal failure, The American journal of physiology 257(3 Pt 2) (1989) F440-5.
[2] Z.Y. Jiang, A.C. Woollard, S.P. Wolff, Hydrogen peroxide production during experimental protein glycation, FEBS letters 268(1) (1990) 69-71.
[3] V.J. Thannickal, B.L. Fanburg, Activation of an H2O2-generating NADH oxidase in human lung fibroblasts by transforming growth factor beta 1, The Journal of biological chemistry 270(51) (1995) 30334-8.
[4] C.T. Andrade, L.A.M. Barros, M.C.P. Lima, E.G. Azero, Purification and characterization of human hemoglobin: effect of the hemolysis conditions, International Journal of Biological Macromolecules 34(4) (2004) 233-240.
[5] J. Schneider, C.J. Reckmeier, Y. Xiong, M. von Seckendorff, A.S. Susha, P. Kasák, A.L. Rogach, Molecular Fluorescence in Citric Acid-Based Carbon Dots, The Journal of Physical Chemistry C 121(3) (2017) 2014-2022.
[6] M. Chen, H. Zhou, X. Liu, T. Yuan, W. Wang, C. Zhao, Y. Zhao, F. Zhou, X. Wang, Z. Xue, T. Yao, C. Xiong, Y. Wu, Single Iron Site Nanozyme for Ultrasensitive Glucose Detection, Small 16(31) (2020) 2002343.
[7] W. Dong, G. Chen, X. Hu, X. Zhang, W. Shi, Z. Fu, Molybdenum disulfides nanoflowers anchoring iron-based metal organic framework: A synergetic catalyst with superior peroxidase-mimicking activity for biosensing, Sensors and Actuators B: Chemical 305 (2020) 127530.
[8] Y. Chen, Q. Yuchi, T. Li, G. Yang, J. Miao, C. Huang, J. Liu, A. Li, Y. Qin, L. Zhang, Precise engineering of ultra-thin Fe2O3 decorated Pt-based nanozymes via atomic layer deposition to switch off undesired activity for enhanced sensing performance, Sensors and Actuators B: Chemical 305 (2020) 127436.
[9] Z. Zhao, Y. Huang, W. Liu, F. Ye, S. Zhao, Immobilized Glucose Oxidase on Boronic Acid-Functionalized Hierarchically Porous MOF as an Integrated Nanozyme for One-Step Glucose Detection, ACS Sustainable Chemistry & Engineering 8(11) (2020) 4481-4488.
[10] J. Xian, Y. Weng, H. Guo, Y. Li, B. Yao, W. Weng, One-pot fabrication of Fe-doped carbon nitride nanoparticles as peroxidase mimetics for H2O2 and glucose detection, Spectrochimica Acta Part A: Molecular and Biomolecular Spectroscopy 215 (2019) 218-224.
[11] Y. Zhong, J. Yang, X. Yin, J. Zheng, N. Lu, M. Zhang, Enhanced synergistic effects from multiple iron oxide nanoparticles encapsulated within nitrogen-doped carbon nanocages for simple and label-free visual detection of blood glucose, Nanotechnology 30(35) (2019) 355501.
[12] X. Yin, P. Liu, X. Xu, J. Pan, X. Li, X. Niu, Breaking the pH limitation of peroxidase-like CoFe2O4 nanozyme via vitriolization for one-step glucose detection at physiological pH, Sensors and Actuators B: Chemical 328 (2021) 129033.
[13] H. Chen, C. Yuan, X. Yang, X. Cheng, A.A. Elzatahry, A. Alghamdi, J. Su, X. He, Y. Deng, Hollow Mesoporous Carbon Nanospheres Loaded with Pt Nanoparticles for Colorimetric Detection of Ascorbic Acid and Glucose, ACS Applied Nano Materials 3(5) (2020) 4586-4598.
[14] W. Zhao, G. Zhang, Y. Du, S. Chen, Y. Fu, F. Xu, X. Xiao, W. Jiang, Q. Ji, Sensitive colorimetric glucose sensor by iron-based nanozymes with controllable Fe valence, Journal of Materials Chemistry B 9(23) (2021) 4726-4734.
[15] Q. Wang, X. Zhang, L. Huang, Z. Zhang, S. Dong, One-Pot Synthesis of Fe3O4 Nanoparticle Loaded 3D Porous Graphene Nanocomposites with Enhanced Nanozyme Activity for Glucose Detection, ACS Applied Materials & Interfaces 9(8) (2017) 7465-7471.
[16] T. Li, P. Hu, J. Li, P. Huang, W. Tong, C. Gao, Enhanced peroxidase-like activity of Fe@PCN-224 nanoparticles and their applications for detection of H2O2and glucose, Colloids and Surfaces A: Physicochemical and Engineering Aspects 577 (2019) 456-463.

[17] M.A. Wahab, S.M.A. Hossain, M.K. Masud, H. Park, A. Ashok, M. Mustapić, M. Kim, D. Patel, M. Shahbazi, M.S.A. Hossain, Y. Yamauchi, Y.V. Kaneti, Nanoarchitectured superparamagnetic iron oxide-doped mesoporous carbon nanozymes for glucose sensing, Sensors and Actuators B: Chemical 366 (2022) 131980.
[18] L. Feng, L. Zhang, S. Chu, S. Zhang, X. Chen, Z. Du, Y. Gong, H. Wang, Controllable doping of Fe atoms into MoS2 nanosheets towards peroxidase-like nanozyme with enhanced catalysis for colorimetric analysis of glucose, Applied Surface Science 583 (2022) 152496.
